# The effects of yoga on student mental health: a randomised controlled trial

**DOI:** 10.1080/21642850.2020.1843466

**Published:** 2020-11-11

**Authors:** Tiril Elstad, Pål Ulleberg, Sandra Klonteig, Jonny Hisdal, Gunvor Marie Dyrdal, Arild Bjorndal

**Affiliations:** a Faculty of Medicine, University of Oslo, Oslo, Norway; b Department of Psychology, University of Oslo, Oslo, Norway; c Cognitive Neuroscience, University of Oslo, Oslo, Norway.; d Department of Vascular Surgery, Oslo University Hospital, Oslo, Norway; e Institute of Clinical Medicine, University of Oslo, Oslo, Norway; f Department of Health Sciences in Gjøvik, NTNU, Trondheim, Norway; g Regional Centre for Child and Adolescent Mental Health in Eastern and Southern Norway (RBUP) & University of Oslo, Oslo, Norway

**Keywords:** Mental health, public health, distress, yoga, sleep

## Abstract

**Background:**

Universities around the world are facing an epidemic of mental distress among their students. The problem is truly a public health issue, affecting many and with serious consequences. The global burden of disease-agenda calls for effective interventions with lasting effects that have the potential to improve the mental health of young adults. In this study we aimed to determine whether yoga, a popular and widely available mind-body practice, can improve student mental health.

**Methods:**

We performed a randomised controlled trial with 202 healthy university students in the Oslo area. The participants were assigned to a yoga group or waitlist control group in a 1:1 ratio by a simple online randomisation program. The intervention group was offered 24 yoga sessions over 12 weeks. Measurements were taken at week 0 (baseline), week 12 (post-intervention), and week 24 (follow-up). The primary outcome was psychological distress assessed by the HSCL-25 questionnaire. Analysis was performed based on the intention to treat-principle.

**Results:**

Between 24 January 2017, and 27 August 2017, we randomly assigned 202 students to a yoga intervention group (*n* = 100), or waitlist control group (*n* = 102). Compared with the control group, the yoga participants demonstrated a significant reduction in distress symptoms both at post-intervention (adjusted difference in the mean change −0.15, 95% CI −0.26 to −0.03, *p* = 0.0110) and follow-up (adjusted difference in the mean change −0.18, 95% CI −0.29 to −0.06, *p* = 0.0025). Sleep quality also improved at post-intervention and follow-up. No adverse events were reported.

**Conclusions:**

Our findings suggest that yoga has a moderately large and lasting effect, at least for some months, reducing symptoms of distress and improving sleep quality among students. Further research should seek ways to enhance the effect, assess an even longer follow-up period, include active control groups, and consider performing similar studies in other cultural settings.

**Trial registration:** ClinicalTrials.gov identifier: NCT04258540.

## Introduction

Student mental health is a rising public health issue that is in need of effective, scalable, and attractive interventions. Universities around the world are faced with an increasing prevalence of mental health problems among their students (Auerbach et al., 2018; Hunt & Eisenberg, 2010). There is a growing number of students worldwide (OECD, 2017), and the student period might be a golden opportunity in which to implement mental health interventions (Hunt & Eisenberg, 2010; Jané-Llopis & Barry, 2005). A high level of distress is associated with decreased well-being, academic impairment, and adverse socioeconomic effects (Bruffaerts et al., 2018). Mental health problems account for nearly half of the disease burden of young adults (Erskine et al., 2015). Help-seeking behaviour at universities has been improving in recent years, and stigma has been reduced (Hunt & Eisenberg, 2010). However, the need for support exceeds the available mental health services at most universities (Auerbach et al., 2018).

Investing in the mental health of young adults will not only generate health-related and economic benefits today, but also for decades to come and for future generations (Patton et al., 2016; World Health Organizaion, 2008). Moreover, traditional one-on-one psychotherapy is costly and may also not be acceptable to first-time users of mental health services (Brown, 2018). Known barriers to help-seeking is that many students view their problems as a normal part of student life and that they want to address their issues on their own (Eisenberg, Speer, & Hunt, 2012). Mental health initiatives should be holistic and emphasise the promotion of mental health, including increased well-being, as well as alleviating mental distress (Winzer, Lindberg, Guldbrandsson, & Sidorchuk, 2018).

Yoga, a popular and readily available mind–body practice, is safe, has a low barrier to entry, and may easily be cost-effective as it is offered to large groups. A standard yoga class varies in style according to the teacher and focus, but is usually composed of physical postures, breathing techniques, and simple meditation exercises, with or without teachings on yoga philosophy. There is a growing amount of research on yoga, but few RCTs have included a student population. One recent meta-analysis assessing the effects of yoga, meditation, or mindfulness on student distress showed moderate effects post-intervention, but most of the 24 included studies were of poor quality (Breedvelt et al., 2019). Eight of the studies investigated the effects of yoga, and only one study included a follow up of more than four weeks. None of the studies reported on safety of the yoga course and none were conducted in Europe. To our knowledge, no RCT with adequate sample size and follow-up beyond the intervention period evaluating the effects of yoga on mental health among healthy students exists.

The aim of this study was therefore to provide a rigorous evaluation of yoga’s effect on student distress and other mental health outcomes among healthy university students. We used both measures of psychological and somatic distress, as well as questionnaires on sleep, mindfulness, and well-being, addressing the broader definition of mental health proposed by the World Health Organisation; ‘A state of well-being in which an individual realizes his or her own abilities, can cope with the normal stresses of life, can work productively and is able to make a contribution to his or her community’ (World Health Organizaion, 2008). We chose a non-clinical population as part of a wider public health strategy to ameliorate distress among a greater number students. We hypothesised that yoga would generate positive changes in both psychological and physiological outcomes and, hence, contribute to increased student mental health.

## Materials and methods

### Prior evidence

On 16 July 2019 we searched PubMed with no language or date restrictions for (students) AND (yoga) AND (mental health OR well-being OR well being OR wellbeing OR stress OR distress OR depression OR anxiety OR fatigue OR sleep problems). Our search identified 222 papers, including systematic reviews, meta-analyses, and randomised controlled trials (RCTs), showing evidence for the effect of yoga programs on a variety of health measures for a broad range of target groups. However, most studies were of poor quality. Systematic reviews warranted more studies on yoga in non-clinical populations, including RCTs, larger studies, longer follow-ups, detailed methods description, and better reporting. We failed to identify any RCT that fulfilled those requirements. This was an update of our search from the planning stage of our study.

### Trial design

The study is a randomised controlled trial including students from public and private universities in Oslo, Norway. The study was approved by the Regional Committee for Medical Research Ethics in South-East Norway in November 2016. The protocol was published on the study web-site before the study started (available at https://yogastudy.tilda.ws/). The study conformed to the CONSORT (Strand, Dalgard, Tambs, & Rognerud, 2003) and TIDieR (Pallesen et al., 2008) checklist which is available as supplementary material.

### Participants

Eligible candidates were university and college students 18 years and older living in the Oslo area. The exclusion criteria were serious mental health diagnoses, recent major life crisis, and having an established, systematic yoga practice during the prior six months. The official student health body, SiO Health*,* distributed flyers to potential candidates. Posters, flyers, and social media campaigns were also used for recruitment. Information brochures and consent forms were managed through email. Written consent was obtained before enrolment in the study.

### Procedures

#### Intervention group

100 students were randomised to the intervention group and offered a yoga course at HiYoga in Oslo. Sessions lasting 1.25 h were offered two times a week for 12 weeks. The class size was kept at approximately 25 students. Students signed an attendance list at the beginning of each class. Two parallel programs were taught each week, and if absent, participants were contacted by the study coordinator to offer a possible rescheduling.

The yoga program was designed for the purpose of this study and based on Ashtanga Vinyasa. It was quite similar to most other yoga programs as it consisted of asanas (yoga postures), pranayama (breathing exercises), and dhyana (meditation), in addition to some yoga philosophy. The physical exercises were designed to promote strength, flexibility, stamina, and balance. The breathing and meditation exercises varied in purpose and style and included, among other practices, ‘deep relaxation’, ‘even breathing’, and ‘sound meditation’. Students were given detailed instructions in class and in handouts and were encouraged to practice at home. A new theme was introduced each week, including breathing, sun-salutations, inversions, and drishti (focused gaze). The classes were taught by three certified yoga instructors with many years of teaching experience, however none had taught this exact sequence and program before. The yoga teachers followed a program schedule and were instructed to teach the same sequences and themes each week to limit differences between the groups.

#### Control group

102 participants were informed about admittance in the study and randomised to the waitlist control group. These students were asked to refrain from yoga during the assessment period, but all other forms of exercise were allowed. Students in the control group were offered the same yoga course after the last follow-up assessment.

#### Assessments

The intervention ran during the spring and fall semesters of 2017. Primary outcome data (psychological distress) were collected at week 0 (baseline), week 12 (post-intervention), and week 24 (follow-up). At each measurement point, participants completed an online questionnaire. Participants who did not complete the questionnaire received two reminder-emails the following week. If information was still missing, the study coordinator contacted participants. All questionnaires were administered, and data collected, through an online portal designed to ensure participants’ anonymity.

Data on heart rate variability (HRV) was only collected in the spring semester at week 0 and week 12 (*N* = 105) because we unfortunately had to return the equipment that was borrowed. Participants received a pulse watch and pulse belt at HiYoga and were instructed to wear the wristband and belt overnight to collect nocturnal HRV-data during sleep. Participants also received a user manual and support contact details, and were asked to refrain from alcohol and drug use during the assessment.

### Outcomes

The outcomes were selected with an overall aim to measure student mental health covering psychological distress, sleep quality, somatic distress, life satisfaction, mental well-being, and mindfullness. Deleted a couple of sentences (/ moved to intro).

The primary outcome was psychological distress, measured using the Hopkins Symptom Checklist (HSCL-25) (Strand et al., 2003). This well-known instrument consists of 25 items on symptoms of depression and anxiety, including shivering, rapid heart beat, feelings of hopelessness, and suicidal thoughts. Each question is rated on a 1 (not at all) to 4 (very much) Likert scale. The mean score of the 25 items range from 1 to 4, a higher score indicating more psychological distress. A cut-off point of 1.75 is often used to designate those with a moderate to high symptom burden. Cronbach’s alpha was 0.91, 0.92, and 0.93 at baseline, post-intervention, and follow-up, respectively.

Sleep was measured using the 6-item Bergen Insomnia Scale (BIS) (Pallesen et al., 2008). Scores range from 0 (no bad nights during the course of a week) to 7 (all nights), with a total score ranging from 0 to 42 (higher scores indicating more troubled sleep and daytime tiredness). Cronbach’s alpha was 0.81 at baseline in the present study.

Somatic distress was measured using heart rate variability (HRV) (Heart rate variability, 1996), which measures activity in the autonomic nervous system and serves as a proxy for distress (Kim, Cheon, Bai, Lee, & Koo, 2018). In this study we used RMSSD (root mean square of successive differences), which is a validated measure of activity in the parasympathetic part of the autonomic nervous system (Heart rate variability, 1996). In general, an increase in RMSSD is associated with increased parasympathetic system activity and less distress. The HRV data were recorded with a pulse watch (Polar V800) and a pulse belt overnight to collect data from a four-hour period during sleep. The pulse watch is a commonly used and recommended device for obtaining measurements of HRV (Heart rate variability, 1996). Representations of the data were individually screened by an expert to prevent data errors due to technical problems.

Life satisfaction was measured using the 5-item Satisfaction With Life Scale (SWLS), which is a widely used instrument for measuring life satisfaction (Pavot & Diener, 2008). Items are scored on a 1- to 7-point Likert scale. The total score is computed by adding all response values (ranging from 5 to 35), with higher scores indicating higher satisfaction. Cronbach’s alpha of SWLS was estimated to be 0.88 at baseline in the present study.

Mental well-being was measured using the 14-item Warwick-Edinburgh Mental Well-Being Scale (WEMWBS). This scale has five response categories (from ‘not at all’ to ‘all the time’) that are added together to produce a total score ranging from 14 to 70. Higher scores indicate higher levels of mental well-being. The scale appears to be a valid and precise instrument for measuring well-being in Norwegian health care patients (Smith, Alves, Knapstad, Haug, & Aaro, 2017). In the current study, Cronbach’s alpha of WEMWBS was estimated to be 0.90 at baseline.

Dispositional mindfulness was measured using the 15-item Mindful Attention Awareness Scale (MAAS) (Brown & Ryan, 2003). Score values on all individual items were added, producing a total score ranging from 15 to 75, with higher scores indicating higher levels of mindfulness. Cronbach’s alpha of MAAS was 0.86 at baseline in the present study.

### Sample size

To enable the detection of an effect of *d* = 0.40 for changes in the HSCL-25, a total sample of 150 participants was needed (Strand et al., 2003), which provided 80% power (two-side *α* = 0.05). This effect size corresponds to a 0.13-point difference in the HSCL-25 when post-intervention and follow-up data are included in the same analysis. Expecting 25% attrition resulted in 200 participants being recruited for this study, who were randomised in a 1:1 fashion to the intervention and the control group.

### Randomisation and masking

Students were given a unique participant number and randomised to either the intervention group or the waitlist control group. The randomisation process was performed by one of the researchers using an online randomisation software program. The only stratifying criterion used was gender, in order to ensure that the relatively few males were equally distributed to intervention and control. Outcome assessors and those performing the statistical analysis were blinded to group assignments.

### Adverse events

Yoga instructors were asked to fill out a form registering date, time, event, cause, and severity, in the case of injuries or other adverse events occurring during class. There was no other control measure of adverse events.

### Statistical methods

To detect changes over time, data were analysed using linear mixed effects regression models (LMM), accounting for data missing at random in the primary and secondary outcome variables. To avoid biased estimates of the treatment effect, the baseline value of the outcome variable of interest was included as a covariate to adjust for baseline differences in the outcome variable between the two groups (Twisk et al., 2018). Time point (post-intervention vs. follow-up) and group (yoga vs. control) were included as dummy-coded fixed effects. We also included the interaction between group and time point as a fixed effect to estimate the treatment effect at the two time points. The results are presented as the mean-adjusted differences in mean scores between the yoga group and control group at each time point, controlled for baseline scores in the outcome variable of interest. Additional LMM analyses using baseline, post-intervention, and follow-up as three timepoints were also performed to compare the two groups in their development in mean scores from baseline to follow-up. These additional analyses are presented in the Appendix. For Heart Rate Variability (HRV) we only collected baseline and post-intervention data. Logistic regression analysis was used to study differences between the intervention group and the control group in the number of participants scoring above the cut-off value of 1.75 on the primary outcome variable HSCL-25 at post-test and at follow-up. Again, baseline scores were included as a covariate.

We confirmed the assumption of the normal distribution of residuals in both the primary outcome (HSCL-25) and all secondary outcomes (SWLS, WEMWBS, MAAS, BIS, and HRV). For all analyses, significance was set at 0.05 (two-sided), and IBM SPSS version 25.0 was used. Information about missing data patterns and sensitivity analyses can be found in the Appendix. No exclusion criteria were set in the data handling other than successful data collection at baseline and at least one of the two following time points.

## Results

**Figure 1.**
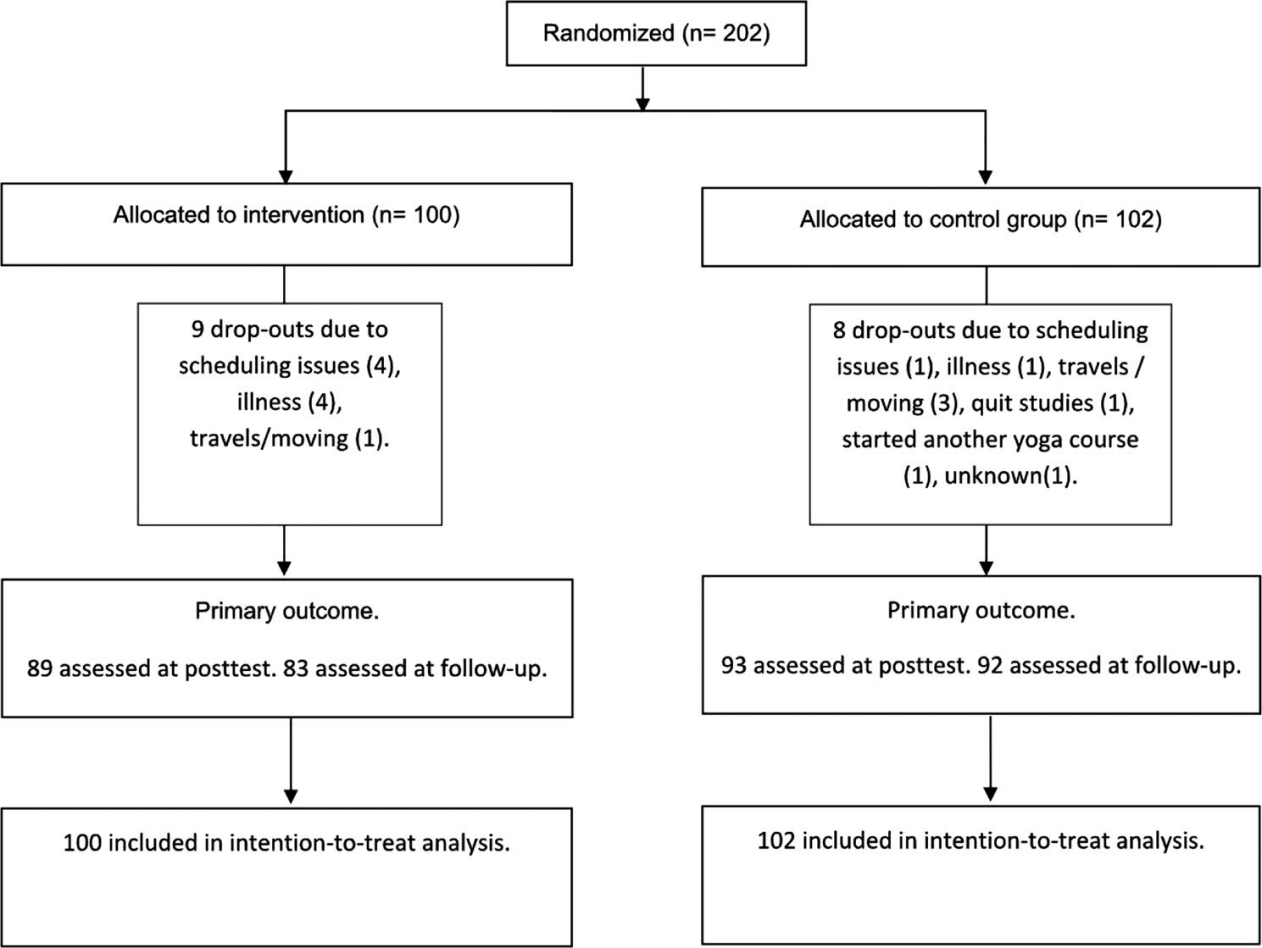
Flow chart.

The 202 participants were randomly assigned to either the yoga intervention group (*n* = 100) or the control group (*n* = 102) (see Figure 1 Flowchart for details). The sample consisted mainly of females (87%), and mean age was 25 years (Table 1). The two groups were well matched at baseline, except for the intervention group having somewhat higher baseline scores on the primary outcome of psychological distress, HSCL-25. The dropout rate was lower than expected (8.4%) and was of approximately equal size in the yoga group and the control group. The baseline score for the primary outcome, HSCL-25, was not related to later dropout (see Appendix). Attendance to the yoga session was high in the intervention group, 91 students (100%) attended at least eight sessions, 78 (84%) participated in at least 20 sessions, and 52 (57%) attended all 24 sessions.

**Table 1.** Baseline characteristics of the sample.

	Control group (*n* = 102)	Yoga group (*n* = 100)
Age (years)	25.00 (4.05)	25.63 (4.11)
Sex		
Male	12 (11.8%)	14 (14.0%)
Female	90 (88.2%)	86 (86.0%)
Primary outcome at baseline		
HSCL-25	1.84 (0.44)	1.92 (0.50)
HSCL-25 cut-off		
Above (>1.75)	54 (52.9%)	60 (60.0%)
Below (≤1.75)	48 (47.1%)	40 (40.0%)
Secondary outcome at baseline		
SWLS	22.61 (5.93)	21.47 (6.04)
WEMWBS	46.15 (8.18)	45.27 (8.07)
MAAS	55.12 (11.88)	53.42 (11.69)
BIS	14.30 (8.38)	16.00 (8.60)
Secondary outcome at baseline*	(*n* = 37)	(*n* = 44)
HRV	63.95 (30.84)	72.36 (36.08)

Notes: Data are the mean (SD) or *n* (%). HSCL-25 = Hopkins Symptoms Checklist 25, SWLS = Satisfaction With Life Scale, WEMWMS = Warwick-Edinburgh Mental Wellbeing Scale, MAAS = Mindful Attention Awareness Scale, BIS = Bergen Insomnia Scale.

*At baseline, 37 participants had valid HRV scores in the control group, and 44 in the participants had valid HRV-scores in the intervention group.

The yoga group showed significant reductions in psychological distress, at both post-intervention and follow-up compared with the control group (Table 2). The effect size of this reduction was −0.32 at post-intervention and −0.38 at follow-up, suggesting a moderate effect of the yoga intervention. An additional analysis showed that only the yoga group had a significant reduction in psychological distress from baseline to post-intervention and from baseline to follow-up (see Appendix). Furthermore, the yoga group had a 41% decrease in risk for scoring above the HSCL-25 cut-off value of 1.75 at post-test compared to the control group (risk ratio 0.59, 95% CI 0.35 to 0.95, *p* = 0.0146), and the corresponding risk reduction was 33% at follow-up (risk ratio 0.67, 95% CI 0.39 to 1.03, *p* = 0.0734, see Appendix).

**Table 2.** Primary outcome results.

	Psychological distress (HSCL-25)			
	Unadjusted mean		Adjusted difference* (95% CI), *d*^†^	*p* value*
	Control	Yoga		
Post-intervention	1.82 (0.46)	1.74 (0.45)	−0.15 (−0.26 to −0.03), −0.32	0.0110
Follow-up	1.78 (0.52)	1.65 (0.45)	−0.18 (−0.29 to −0.06), −0.38	0.0025

Notes: Data are the mean (SD). At post-intervention, 93 participants were in the control group, and 89 participants were in the yoga group. At follow-up, 92 participants were in the control group, and 83 participants were in the yoga group. HSCL-25 = Hopkins Symptoms Checklist 25.

*Linear mixed effects model adjusted for baseline score on HSCL-25, time and interaction of time with randomisation, including a random intercept for participants. Covariance matrix of within-participant measurements was variance components.

^†^ *d* is standardised effect size (Cohen’s *d*).

The effect of the intervention was also estimated using two types of sensitivity analyses (see Appendix). Including only participants having valid scores on both post-intervention and follow-up on HSCL-25 gave gave only trivial differences from the main analysis. Likewise, intention-to-treat analysis using multiple imputation for missing values on HSCL-25 also gave the same results as the main analysis. As the attendance to the yoga sessions in general was very high, it was not meaningful to estimate whether the effect of the yoga intervention was different for participants with low adherence.

The results also suggested that the yoga intervention had favourable effects upon the secondary outcome measures at post-intervention (Table 3). The yoga group showed moderate improvements in well-being (SWLS and WEMWBS), mindfulness (MAAS) as well as a moderate reduction in problems with insomnia (BIS) at post-intervention as opposed to the control group. Scores on secondary outcomes were sustained at follow-up (except for MAAS) for the yoga group, but due to improvements from post-intervention to follow-up in SWLS, WEMWBS and MAAS in the control group, differences were no longer significant. These results suggest that the yoga intervention had only short-time effects upon well-being and mindfulness. Still, the results suggested a long term reduction in insomina in the yoga group, while problems with insomnia were relatively unchanged at follow-up in the control group. The mean score on HRV was slightly higher in the yoga group at post-intervention, but the difference was not statistically significant (*p* = 0.22, Table 4). Thus, no evidence for an effect on heart rate variability was found. Two types of sensitivity analyses (per-protocol and intention-to-treat) gave the same conclusion as the main analysis regarding all secondary outcomes (Appendix).

**Table 3.** Secondary outcome results.

	Unadjusted mean		Adjusted difference* (95% CI), *d*^†^	*p* value*
	Control	Yoga		
SWLS				
Post-intervention	22.27 (6.13)	23.43 (5.40)	2.13 (0.83 to 3.43), 0.36	0.0014
Follow-up	22.86 (6.75)	23.40 (5.84)	1.29 (−0.03 to 2.61), 0.22	0.0553
WEMWBS				
Post-intervention	45.52 (8.87)	47.92 (7.69)	2.95 (0.84 to 5.06), 0.36	0.0063
Follow-up	47.15 (9.84)	47.31 (8.81)	0.56 (−1.58 to 2.71), 0.07	0.6051
MAAS				
Post-intervention	56.59 (10.95)	59.76 (10.68)	4.40 (1.70 to 7.10), 0.37	0.0015
Follow-up	58.32 (11.37)	58.11 (11.63)	0.76 (−1.98 to 3.49), 0.06	0.5853
BIS				
Post-intervention	14.34 (8.38)	11.98 (7.33)	−3.45 (−5.39 to −1.52), −0.41	0.0005
Follow-up	13.17 (8.59)	11.64 (6.85)	−2.52 (−4.49 to −0.56), −0.30	0.0121

Notes: Data are the mean (SD). At post-intervention, 93 participants were in the control group, and 89 participants were in the yoga group. At follow-up, 92 participants were in the control group, and 83 participants were in the yoga group. SWLS = Satisfaction With Life Scale, WEMWMS = Warwick-Edinburgh Mental Wellbeing Scale, MAAS = Mindful Attention Awareness Scale, BIS = Bergen Insomnia Scale.

*Linear mixed effects model adjusted for baseline score, time and interaction of time with randomisation, including a random intercept for participants. Covariance matrix of within-participant measurements was variance components.

^†^ *d* is standardised effect size (Cohen’s *d*).

**Table 4.** Secondary outcome HRV results.

	Heart rate variability (HRV), measured through the RMSSD			
	Unadjusted mean		Adjusted difference* (95% CI), *d*^†^	*p* value*
	Control	Yoga		
Post-intervention	69.87 (34.70)	80.60 (41.69)	9.61 (−5.80 to 25.02), 0.25	0.2194

Notes: Data are the mean (SD). At baseline, 37 participants were in the control group, and 44 participants were in the yoga group. At post-intervention, 38 participants were in the control group, and 39 participants were in the yoga group. HRV = Heart rate variability.

*Linear mixed effects model with time (baseline vs post intervention), group and interaction of time with group, including a random intercept for participants. Covariance matrix of within-participant measurements was variance components.

^†^ *d* is standardised effect size (Cohen’s *d*).

No adverse effects of yoga were reported.

## Discussion

Yoga twice a week for three months decreased psychological distress among university students in this study, and the effects lasted beyond the yoga course period. At baseline 60% of the participants in the yoga group had a HSCL-25 score above a cut-off value of 1.75, implying that over the past two weeks, many participants had experienced moderate or serious symptoms of distress. After the yoga course 39% were above this cut-off value, and 34% at the three-month follow-up (Appendix).

An effect size of 0.38 is somewhat higher than that found in the literature for a variety of mental health interventions in the general student population (Harrer et al., 2019; Winzer et al., 2018). Few solid studies exist on the effects of yoga specifically on student mental health. One recent meta-analysis on yoga, meditation, or mindfulness interventions among students found a similar effect size on depression, anxiety, and stress, however, relatively poor quality of included RCTs, including small samples, limits the trust placed in conclusions (Breedvelt et al., 2019). We have identified another RCT on yoga and mindfulness including students diagnosed with depression or anxiety, that reported a moderate effect size (Falsafi, 2016). Intervention studies among students diagnosed with mental illness report similar or somewhat higher effect sizes (Bailey, Hetrick, Rosenbaum, Purcell, & Parker, 2018; Cuijpers et al., 2016; de Vibe et al., 2017).

Additionally, our sleep measures revealed a significant, moderately positive effect of yoga in the experimental group at follow-up compared to the control group. Sleep problems are prevalent in the student population (Jiang et al., 2015), and there is an established two-way association between mental distress and sleep (Freeman et al., 2017). Sleep problems are also correlated with worse academic performance (Phillips et al., 2017), and are seen as a marker of more severe mental problems among students.

No clear conclusion regarding the effects of yoga on heart rate variability (HRV) exists in the literature, but studies are of low quality (Posadzki, Kuzdzal, Lee, & Ernst, 2015; Tyagi & Cohen, 2016). In this paper, we have overcome some of those obstacles, i.e. a relatively large sample, a four-hour nocturnal HRV test in which each individual serve as his or her own control, and a detailed methods description. Yet, no effects on HRV was found in our study. Moreover, supplementary analyses in the whole sample revealed no significant correlations between HRV and psychological distress, sleep, or well-being. While HRV is characterised as a potential marker of distress (Heart rate variability, 1996), and findings are heterogenous, we would argue that a critical review is necessitated.

In addition to psychological and somatic distress, this study assessed positive aspects of student mental health. The two well-being measures and the mindfulness questionnaire revealed significant and moderate effects at post-intervention in the yoga group compared with the control group, as expected. At follow-up, the control group had improved, demonstrating how perceived mental health and well-being fluctuate. Our findings add to a line of contradicting conclusions in the literature (Bolier et al., 2013; Harrer et al., 2019; White, Uttl, & Holder, 2019).

As illustrated by the relatively high baseline distress scores, many students struggle. A large, ongoing survey among students in Norway likewise reported a high average score of 1.77 on the same HSCL-25 scale in 2018 based on information from 50,000 students (Sivertsen, Råkil, Munkvik, & Lønning, 2019). Average scores were up from 1.55 in 2010 and 1.64 in 2014. Particularly worrying perhaps, was the trend in serious mental health problems (HSCL-score ≥2.0); rising from 16% (2010) via 21% (2014) to 29% (2018). The high proportion of women in our sample contributes to the high baseline values since women, compared to men, report twice the level of psychological distress (Sivertsen et al., 2019). Similarly high levels of mental distress among students have been reported from many countries, i.e. Australia, Canada, and the US (Dyrbye, Thomas, & Shanafelt, 2006; Stefan Cvetkovski & Jorm, 2012). While all studies on student mental health carry the risk of including a slightly biased sample of youth in need of support, the substantial rise over such a short time period is worrying and may point to external factors like increased academic and social pressure, as well as higher expectations for oneself and one’s life due to social media and other influences. Less stigma and more focus among youth on mental health issues could also be possible explanations.

This study has several limitations that should be addressed. It was the first time the three yoga instructors taught this exact program; some were therefore facilitating new breathing exercises (pranayama) and others were required to teach and adjust new poses (asana). However, all of the instructors were certified and had several years of experience, including teaching new content and sequences. There might be room for improvement of both the program itself and of the teaching of the program. We ended up recruiting a majority of females and of medical students from the University of Oslo, meaning that the study is not entirely representative of the whole student body. This study included a formal assessment plan of injuries, however, as instructors might be biased assessors, future studies should include a second control measure of injuries. Future research might also include an active control group to provide direct comparisons of effects with other approaches, as well as investigate the effects of yoga among students across cultures, nations, and disciplines. Furthermore, studies should ideally have even longer follow-up.

Student mental health is an important public health issue concerning millions of young people all over the world. The burden of mental distress is of such a magnitude that a public health strategy must be applied – improved access to referral-based treatments will not be enough. The high prevalence figures should also lead to a broad debate on how to structure university studies in order to promote mental health. Universities should plan for effective, low-cost, popular interventions relevant for all students, as well as providing easily available counselling to help alleviate distress and improve sleep. Our study suggests that yoga may be an option to alleviate symptoms of depression, anxiety and insomnia among students, and should therefore be considered by university policy makers as part of a whole university approach.

## Conclusions

This study aimed to investigate the effect of yoga on student distress, sleep, mindfulness, and well-being by conducting a methodologically robust RCT. For students in need of readily available and flexible mental health support, yoga may be an appealing option. We found that yoga reduced psychological distress and sleep problems among the students with effects leasting three months beyond the active yoga course period. Our results indicate that yoga might be a moderately effective intervention in alleviating student mental distress. There is a dire need for universities to address mental health issues among students and it seems reasonable to try yoga as a part of a wider strategy to support students through these formative years.

## Supplementary Material

Supplemental Material

Supplemental Material

Supplemental Material

## Data Availability

Data sharing statement is available as a separate attachment.

## Trial registration

The study was retrospectively registered on clinicaltrials.gov, identifyer number NCT04258540, available at https://clinicaltrials.gov/ct2/show/NCT04258540?term=elstad&draw=2&rank=1.
