# Supplementary material for: The effects of yoga on student mental health: a randomised controlled trial
Source: Health Psychol Behav Med. 2020 Nov 11;8(1):573–86. doi: 10.1080/21642850.2020.1843466 (PMC8114365; doi:10.1080/21642850.2020.1843466)
Supplement: Supplemental Material [file RHPB_A_1843466_SM3183.docx]

**Supplementary Appendix**

**1.INTRODUCTION**

This document presents sensitivity analyses of the primary and secondary outcome used in the randomised controlled trial to evaluate the attendance of yoga sessions versus treatment as usual.

- 1. **Software employed**

All analyses were carried out using the software IBM SPSS Statistics version 25·0.

**2. METHODS**

**2.1 Background information**

Students are a large and increasingly vulnerable population in terms of experiencing adverse symptoms of distress. The global burden of disease agenda calls for effective and lasting interventions that have the potential to improve the mental health of young adults.

**2.2. Study design**

The Oslo Yoga Science Study is a single blinded student randomised control trial. The sample consists of 202 university students recruited and randomised to either participate in 12 weeks of yoga sessions (2 sessions each week, 24 sessions in total), or a waiting list for the yoga course at a 1:1 ratio. The recruitment of participants started on 24. January 2017 and ended 27. August 2017.

**2.3 Objectives**

Primary objective

- To determine whether participating in yoga sessions over a 12-week period can reduce symptoms of psychological distress in a sample of college and university students by the end of treatment (both at the end of treatment and at follow-up).

Secondary objective

- To determine whether participating in yoga sessions over a 12-week period can increase life satisfaction, mental well-being, and mindfulness, and reduce symptoms of insomnia and somatic distress in a sample of college and university students by the end of treatment.

**2.4 Target population**

College or university students living in the Oslo area in Norway.

Inclusion criteria

- Age above 18
- University or college student living in the Oslo area
- Wanting to learn yoga
- Believe that they can complete the study

Exclusion criteria

- No serious mental health diagnoses
- No recent major life crisis
- No systematic yoga practices the last six months

**2.5 Intervention**

*Intervention group*

Two times a week (1.25 hr x 2) for 12 weeks, the students in the intervention group were offered a yoga course at HiYoga in Oslo with three certified Yoga Alliance instructors teaching the same yoga program. Yoga class size was kept at about 25 students. The participants were allocated to a random time slot. Before every yoga class, students were asked to sign an attendance list. If absent, participants were contacted by a course coordinator in order to arrange for rescheduling of time slots, if applicable.

The yoga course was designed for the purpose of this study and consisted of (i) asanas (yoga postures), (ii) pranayama (breathing exercises), and (iii) dhyana (meditation), in addition to some yoga philosophy and handouts. The program was based on the Hatha yoga tradition, borrowing a great number of postures from The Ashtanga Primary Series and Vinyasa flow sequences. The physical exercises were designed to promote strength, flexibility, stamina, and balance. The breathing- and meditation exercises varied in purpose and style and included among other practices “deep relaxation”, “even breathing”, and “sound meditation”. Students were given detailed instructions in class and in handouts, and were encouraged to practice at home. A new theme was introduced each week, including, but not limited to breathing, sun salutations, inversions, and drishti (focused gaze).

*Control group*

The participants in the waitlist control group were asked to refrain from yoga during the assessment period, however, all other forms of exercise were allowed. They were offered the same yoga course after the last follow-up assessment.

**2.6 Outcome measures**

The primary outcome of interest in this study was psychological distress, measured through The Hopkins Symptom Checklist (HSCL-25) questionnaire. The secondary outcomes life satisfaction, mental well-being, mindfulness, and insomnia were measured using Satisfaction with Life Scale (SWLS), Warwick-Edinburg Mental Well-Being Scale (WEMWBS), Mindful Attention Awareness Scale (MAAS), and Bergen Insomnia Scale (BIS), respectively. All of the above measures were self-report questionnaires. In contrast, the secondary outcome somatic distress was measured through Heart Rate Variability (HRV).

For logistic matters the study was organized in two sessions; spring round and fall round of 2017. The primary outcome assessments of psychological distress and the assessment of the secondary outcomes; life satisfaction, mental well-being, mindfulness, and insomnia were carried out in weeks 0 (T1, baseline), 12 (T2, post-intervention), and 24 (T3, follow-up). At each measurement point every participant logged on to and completed an online questionnaire. If participants missed the deadline, they received two notification emails the following week. Finally, if they still had not answered after the reminders, they were contacted by phone/ email by a study coordinator.

Assessments for the secondary outcome, somatic distress, were undertaken only in the spring round in weeks 0 (T1, baseline) and 12 (T2, post-intervention). All 202 participants picked up a pulse watch and -belt at HiYoga which were to be returned the following day. They received a user manual along with support contact detail and were asked to refrain from alcohol and drugs during the assessment. The participants were instructed to wear the wristband and belt overnight in order to collect nocturnal HRV data during sleep.

*Primary outcome, HSCL-25.*

Psychological distress was measured by participants’ score on a shortened version of The Hopkins Symptom Checklist (HSCL); The Hopkins Symptom Checklist-25 (HSCL-25). HSCL has shown strong validity, reliability, and consistency as a measure for present and change in a variety of psychological symptoms. In the present study, Cronbach’s α was 0·91, 0·92, and 0·93 at baseline, posttest, and follow-up, respectively. The shortened version extract 25-items from the HSCL with a special focus on symptoms of depression and anxiety, commonly referred to as distress. HSCL-25 is an accurate and practical representative of the full-scale version for this specific group of symptoms.^1^ HSCL-25 is a well-known scale used in national Norwegian studies^2^, The Journal of the American Medical Association (JAMA), and described as a useful tool to measure general distress in other populations.^3^ There are four scores in the scale that extend between 1=”Not at all” and 4=”Extremely”, where the number positively increases with the degree of perceived symptom burden. To calculate the average score, the total score is divided by the number of items answered.^2^ The clinical cut off point of 1·75 traditionally indicates a severe symptom burden.^4^ Others, and this study, have chosen a more conservative interpretation, in which a score between 1·75 and 2 indicates moderate symptoms of distress and above 2 a more serious and persistent symptom burden.^5^ The HSCL-25 questionnaire was administered through the university’s online portal. All data was exported directly to SPSS. The online portal has been designed to ensure participants’ anonymity during data collections.

*Secondary outcomes*

*Life Satisfaction*

Life satisfaction was measured using the 5 item Satisfaction With Life Scale (SWLS), which is the most well-known and widely used instrument for measuring life satisfaction.^6^ Scale scores range from 5 to 35, high scores indicates higher life satisfaction. Cronbach´s alpha for the scale was 0·88 at baseline in the present study.

*Mental Well-Being*

Mental well-being was measured using the 14-item Warwick-Edinburg Mental Well-Being Scale (WEMWBS). Scores range from 14 to 70, high scores indicate higher levels of mental well-being. The scale is found to have good psychometric properties and appear to be a valid and precise instrument for measuring well-being in a Norwegian population.^7^ Cronbach´s alpha of the scale was estimated to be 0·90 at baseline in the present study.

*Mindfulness*

Dispositional mindfulness was measured using the 15-item Mindful Attention Awareness Scale, MAAS.^8^ Scale score ranges from 15 to 75, high scores indicate higher mindfulness. The Cronbach´s alpha of the scale was estimated to be 0·86 at baseline in the present study.

*Insomnia*

Insomnia was measured using the 6-item Bergen Insomnia Scale.^9^ The respondents are asked to rate how many days they experience six different problems with sleeping during a week, ranging from 0 days to 7 days. Scores range from 0 to 42, high scores indicate more insomnia/problems with sleeping. The Cronbach´s alpha of the scale was 0·81 at baseline in the present study.

*HRV*

Somatic distress was measured through heart rate variability (HRV), a measurement of the time-interval between successive heartbeats, called R-R intervals.^10^ HRV is commonly used to measure activity in the autonomic nervous system; increased parasympathetic activity is associated with a rise in HRV, while increased sympathetic activity has the opposite effect.^11^ Autonomic imbalance has been suggested to be a marker of distress.^12^ In this study we used the HRV measure RMSSD (The Root Mean Square of Successive Differences), which is the square root of the average of the squared differences between successive R-R intervals. RMSSD is a measurement of parasympathetic activity.^10^ The hypothesis is that an increase in RMSSD is associated with more activity in the parasympathetic system and thus less distress. The HRV data was recorded on Polar V800 pulse watches and belts overnight when the participants were sleeping, collecting nocturnal HRV. Pulse watch is a common and recommended device for measurements of HRV.^10^ The Polar V800 watch specifically has been validated from an objective source.^13^ The data was individually downloaded from the Polar watch and synchronized with PolarFlow. A time interval of 4 hours during night was set for the nocturnal data collection. Representations of the data were individually screened by an expert to prevent data errors due to technical problems. Data was exported from the software PolarFlow to SPSS.

**2.7 Sample size**

Based on the standard deviation in a previous study of the HSCL-25 (SD=0·33)^2^, a total sample of 150 participants would provide 80 % power (two-side α=0·05) to detect an effect of d=0·40 for change in HSCL-25 (corresponding to a 0·13 points difference on the HSCL-25) including both post-intervention and follow-up in the same analysis. Taking into account 25 % attrition, we aimed to recruit about 200 participants.

**2.8. Data cleaning**

All questionnaire items were filled in using an online questionnaire, a data-collection method ensuring that each score was valid. Composite scores were calculated for the primary outcome.

The heart rate variability data was recorded on Polar V800 pulse watches. The data was individually downloaded from the Polar watch and synchronized with PolarFlow. Representations of the data were individually screened by an expert to prevent data errors due to technical problems.

All complete case data was included in the analysis with treatment set as randomised. Missing data was considered in a sensitivity analysis.

**3. RESULTS**

**3.1 Baseline characteristics of the participants**

The participants were randomised to either the yoga group or the intervention group with a waiting list an allocation ration of 1:1. The sample consisted of mainly females (87%) and the mean age was about 25 years (table 1). The two randomised groups were well matched at baseline (table 1), but the intervention group had slightly higher baseline scores on both psychological distress, HSCL-25, and Heart Rate Variability (HRV).

**TABLE 1 BASELINE CHARACTERISTICS OF THE INTENTION-TO-TREAT POPULATION**

|  | Control (n =102) | Treatment (n =100) |
| --- | --- | --- |
|  |  |  |
| Age (years) | 25·00 (4·05) | 25·63 (4·11) |
| Sex |  |  |
| Male | 12 (11·8%) | 14 (14·0%) |
| Female | 90 (88·2%) | 86 (86·0%) |
|  |  |  |
| Primary outcome at baseline |  |  |
| HSCL-25 | 1·84 (0·44) | 1·92 (0·50) |
|  |  |  |
| HSCL-25 Cut-off |  |  |
| Above (>1·75) | 54 (52·9%) | 60 (60·0%) |
| Below (≤ 1·75) | 48 (47·1%) | 40 (40·0%) |
|  |  |  |
| Secondary outcome at baseline |  |  |
| SWLS | 22·61 (5·93) | 21·47 (6·04) |
| WEMWBS | 46·15 (8·18) | 45·27 (8·07) |
| MAAS | 55·12 (11·88) | 53·42 (11·69) |
| BIS | 14·30 (8·38) | 16·00 (8·60) |
|  |  |  |
| Secondary outcome at baseline* | (n = 37) | (n = 44) |
| HRV | 63·95 (30·84) | 72·36 (36·08) |
|  |  |  |
| Data are mean (SD) or n (%). HSCL-25=Hopkins Symptoms Checklist 25, SWLS= Satisfaction With Life Scale, WEMWMS= Warwick-Edinburgh Mental Wellbeing Scale, MAAS = Mindful Attention Awareness Scale, BIS=Bergen Insomnia Scale. *At baseline, 37 participants had valid scores on HRV in the control group, and 44 in the participants had valid HRV-scores in the intervention group. | | |

- 1. **Primary analyses**

**3.2.1 Primary outcome**

The Hopkins Symptom Checklist (HSCL-25) is a composite score based upon 25 items. The score can range from 1 to 4, with higher values indicating more psychological distress. Assumptions of normality of the scores on the primary outcome and of the residuals estimated on the basis of a linear mixed effect model was checked using graphical methods. The distribution of the scores on the primary outcome was found to be skewed to the right at both post-test and follow-up (Figure 1) but regarded as adequately normal distributed and thus appropriate to fit the linear mixed model. More importantly, the residuals were approximately normally distributed (Figure 2).


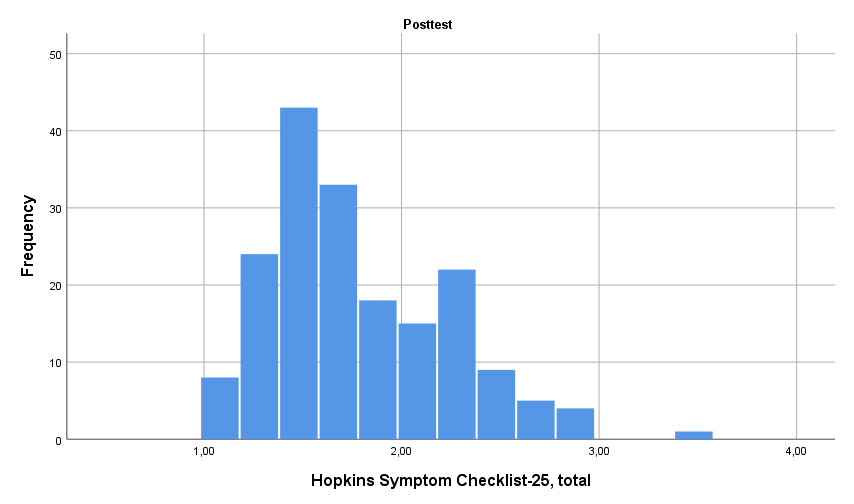


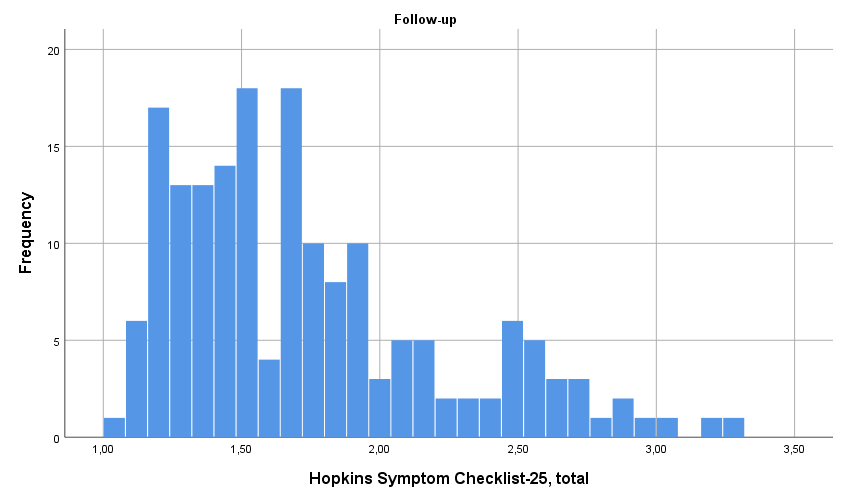


**FIGURE 1. HISTOGRAMS OF HSCL-25 SCORES AT POSTTTEST AND FOLLOW-UP**


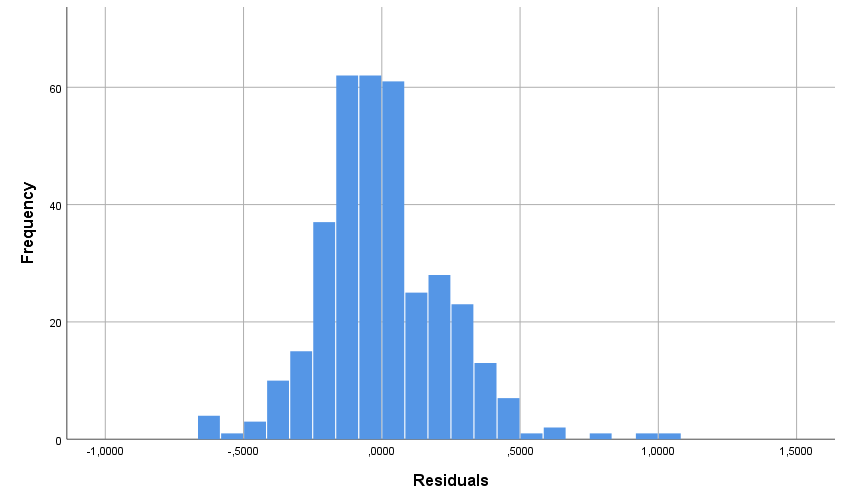


**FIGURE 2. HISTOGRAMS OF HSCL-25 RESIDUALS.**

Analyses using linear mixed effects model showed that the intervention group had significantly reductions in the primary outcome psychological distress, on both the post-intervention (p=0·0110) and the follow-up (p=0·0025) compared with the control group (table 2). The reduction in psychological distress was regarded as moderate (table 2). The difference between treatment effect at post-intervention and follow-up, estimated by the interaction between time and randomization, was small and non-significant (p=0·6075, table 2).

The proportion of participants scoring above the cut-off on psychological distress was lower in the intervention group compared to the control group. Table 3 shows that the intervention group had a 41% decrease in risk for scoring above cut-off at posttest compared to the control group (p = 0.0146) , and the corresponding risk reduction was 33% at follow-up. The reduction in risk at follow-up was however not statistically significant (p=0.0743).

**TABLE 2. PRIMARY OUTCOME RESULTS**

|  | **Psychological distress (HSCL-25)** | | | |
| --- | --- | --- | --- | --- |
|  | Unadjusted mean | | Adjusted difference* (95% CI), d† | p value* |
|  | Control | Treatment |  |  |
| Post-intervention | 1·82 (0·46) | 1·74  (0·45) | -0·15 (-0·26 to -0·03), -0·32 | 0·0110 |
| Follow-up | 1·78 (0·52) | 1·65  (0·45) | -0·18 (-0·29 to -0·06), -0·38 | 0·0025 |
| Time x randomization |  |  | -0·03 (-0·15 to 0·09), -0·06 | 0·6075 |
| Data are mean (SD). At post-intervention, 93 participants were in the control group and 89 participants were in the treatment group. At follow-up, 92 participants were in the control group and 83 participants were in the treatment group. HSCL-25=Hopkins Symptoms Checklist 25. *Linear mixed effects model adjusted for baseline score on HSCL-25, time and interaction of time with randomisation, and including a random intercept for participants. Covariance matrix of within subject measurements was variance components. †d is standardised effect size (Cohen’s d). | | | | |

**TABLE 3. PRIMARY OUTCOME RESULTS BASED UPON CUT-OFF SCORES.**

|  | **Psychological distress (HSCL-25)** | | | | |
| --- | --- | --- | --- | --- | --- |
|  | Above cut-off (1·75) | | Adjusted odds ratio^a^ (95% CI) | Adjusted risk ratio^b^  (95% CI) | p value^a^ |
|  | Control | Treatment |  |  |  |
| Post-intervention | 47 (50·5%) | 35 (39·3%) | 0·42 (0·21 to 0·84) | 0·59 (0·35 to 0·91) | 0·0146 |
| Follow-up | 39 (42·2%) | 28 (33·7%) | 0·54 (0·27 to 1·06) | 0·67 (0·39 to 1·03) | 0·0743 |
| Data are N (%). At post-intervention, 93 participants were in the control group and 89 participants were in the treatment group. At follow-up, 92 participants were in the control group and 83 participants were in the treatment group. HSCL-25=Hopkins Symptoms Checklist 25. ^a^Logistic regression model using adjusted for number of participants within each group scoring under or above cut-off score on HSCL-25. ^b^Risk ratio estimated from adjusted odds ratio. | | | | | |

- 1. **Secondary analyses**
     1. **Secondary outcomes SWLS, WEMWBS, MAAS, and BIS**

**TABLE 4. SECONDARY OUTCOME RESULTS**

|  | Unadjusted mean | | Adjusted difference* (95% CI), d† | p value* |
| --- | --- | --- | --- | --- |
|  | Control | Treatment |  |  |
| SWLS |  |  |  |  |
| Post-intervention | 22.27 (6·13) | 23·43 (5·40) | 2·13 (0·83 to 3·43), 0·36 | 0·0014 |
| Follow-up | 22·86 (6·75) | 23·40 (5·84) | 1·29 (-0·03 to 2·61), 0·22 | 0·0553 |
| Time x randomization |  |  | -0·84 (-2·25 to 0·57), -0·14 | 0·2407 |
| WEMWBS |  |  |  |  |
| Post-intervention | 45·52 (8·87) | 47·92 (7·69) | 2·95 (0·84 to 5·06), 0·36 | 0·0063 |
| Follow-up | 47·15 (9·84) | 47·31 (8·81) | 0·56 (-1·58 to 2·71), 0·07 | 0·6051 |
| Time x randomization |  |  | -2·39 (-4·70 to -0·07), -0·29 | 0·0434 |
| MAAS |  |  |  |  |
| Post-intervention | 56·59 (10·95) | 59·76 (10·68) | 4·40 (1·70 to 7·10), 0·37 | 0·0015 |
| Follow-up | 58·32 (11·37) | 58·11 (11·63) | 0·76 (-1·98 to 3·49), 0·06 | 0·5853 |
| Time x randomization |  |  | -3·64 (-6·23 to -1·06), -0·31 | 0·0060 |
| BIS |  |  |  |  |
| Post-intervention | 14·34 (8·38) | 11·98 (7·33) | -3·45 (-5·39 to -1·52), -0·41 | 0·0005 |
| Follow-up | 13·17 (8·59) | 11·64 (6·85) | -2·52 (-4·49 to -0·56), -0·30 | 0·0121 |
| Time x randomization |  |  | 0·93 (-0·99 to 2·86), 0·11 | 0·3407 |
| Data are mean (SD). At post-intervention, 93 participants were in the control group and 89 participants were in the treatment group. At follow-up, 92 participants were in the control group and 83 participants were in the treatment group. SWLS= Satisfaction With Life Scale, WEMWMS= Warwick-Edinburgh Mental Wellbeing Scale, MAAS = Mindful Attention Awareness Scale, BIS=Bergen Insomnia Scale. *Linear mixed effects model adjusted for baseline score, time and interaction of time with randomisation, and including a random intercept for participants. Covariance matrix of within subject measurements was variance components. †d is standardised effect size (Cohen’s d). | | | | |

*Life Satisfaction*

Life satisfaction was measured using the 5 item Satisfaction With Life Scale (SWLS), which is the most well-known and widely used instrument for measuring life satisfaction.^6^ Scale scores can range from 5 to 35, high scores indicates higher life satisfaction

The distribution of the scores on the SWLS was found to be somewhat skewed to the right at both post-test and follow-up (Figure 3) but regarded as adequately normal distributed to fit the linear mixed model. The residuals were approximately normally distributed (Figure 4).

A linear mixed effects model showed that the yoga intervention seem to lead to moderate improvement in SWLS in the treatment group compared to the control group at post intervention (d=0·36, p=0·0014, but this intervention effect was reduced and became non-significant at follow-up (table 4).


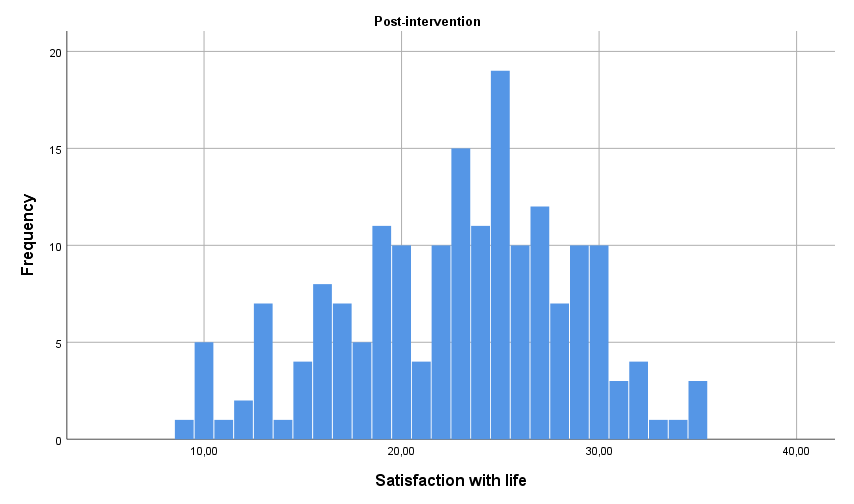


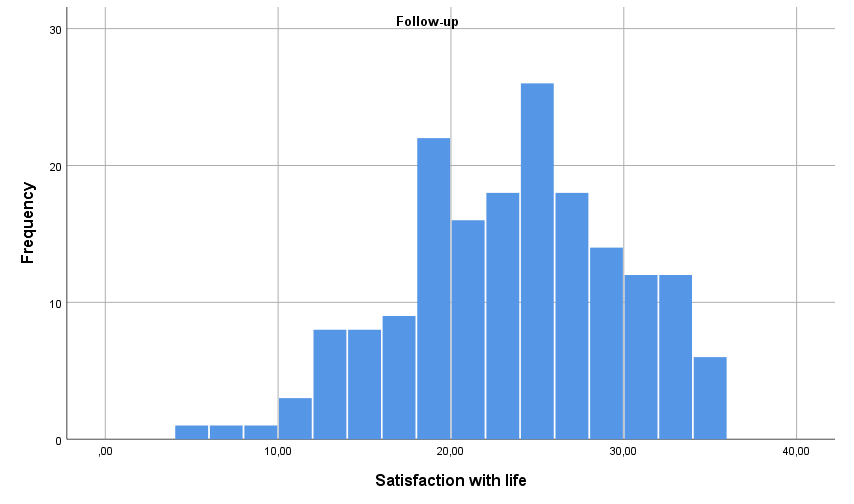


**FIGURE 3. HISTOGRAMS OF SWLS SCORES AT POST-INTERVENTION AND FOLLOW-UP**

**
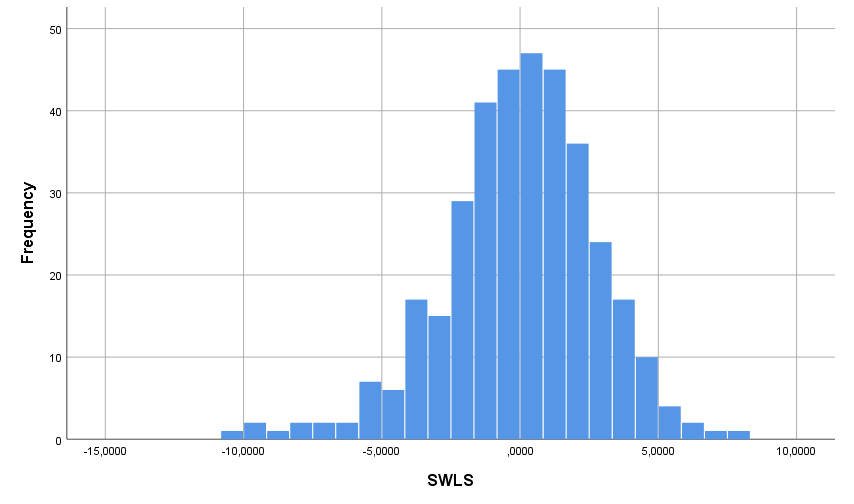
**

**FIGURE 4. HISTOGRAMS OF SWLS RESIDUALS.**

*Mental Well-Being*

Mental well-being was measured using the 14-item Warwick-Edinburg Mental Well-Being Scale (WEMWBS). Scores can range from 14 to 70, high scores indicate higher levels of mental well-being.

The distribution of the scores on the WEMWBS was found to be approximately normal distributed at both post-test and follow-up (Figure 5), and thus regarded as adequately normal distributed to fit the linear mixed model. The residuals were also approximately normally distributed (Figure 6).

A linear mixed effects model showed that the yoga intervention seem to lead to moderate improvement in WEMWBS in the treatment group compared to the control group at post intervention (d=0·36, p=0·0063), but this intervention effect was considerably reduced and became non-significant at follow-up (table 4). There was a significant reduction in treatment effect between post-intervention and follow-up, estimated by the interaction between time and randomization, (p=0·0434, table 4)


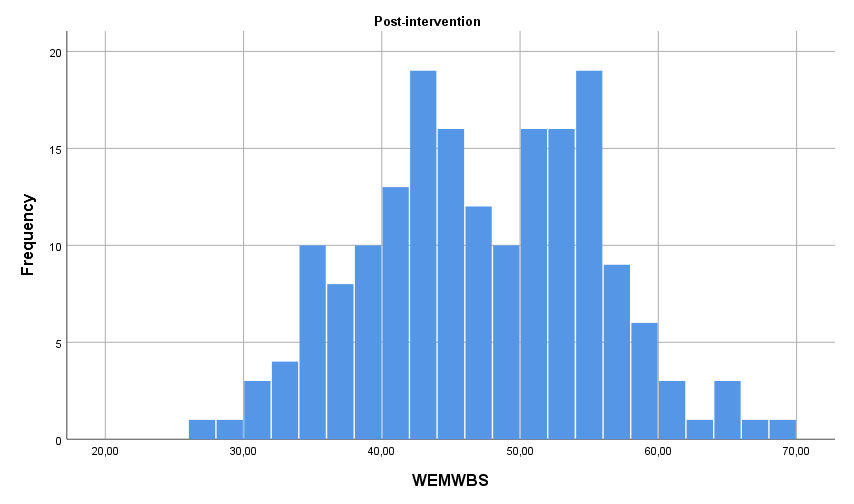


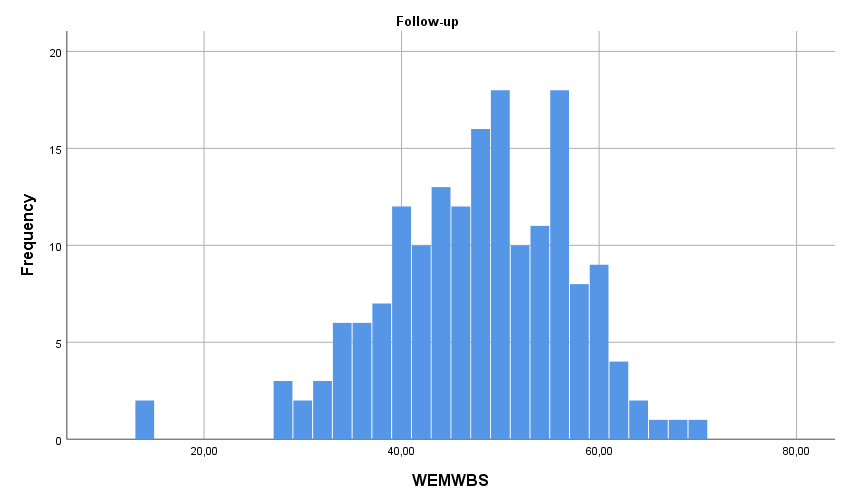


**FIGURE 5. HISTOGRAMS OF WEMWBS SCORES AT POST-INTERVENTION AND FOLLOW-UP**


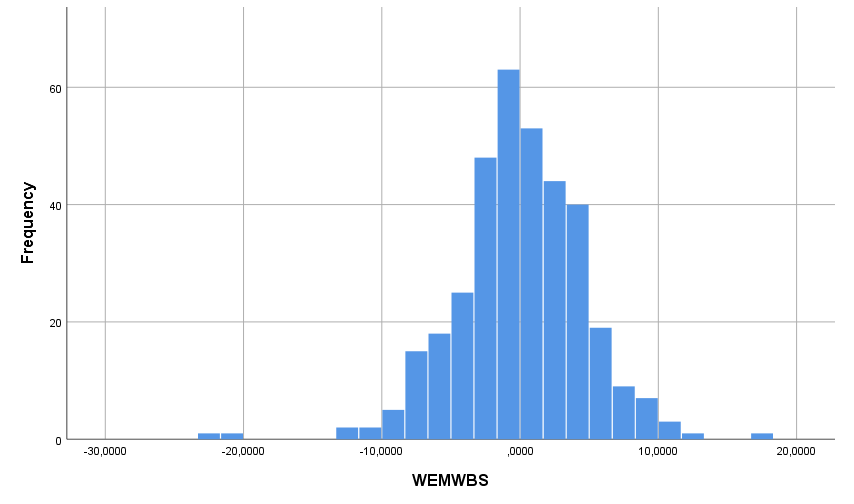


**FIGURE 6 . HISTOGRAMS OF WEMWBS RESIDUALS.**

*Mindfulness*

Dispositional mindfulness was measured using the 15-item Mindful Attention Awareness Scale, MAAS.^8^ Scale score ranges from 15 to 75, high scores indicate higher mindfulness.

The distribution of the scores on the MAAS was found to be somewhat skewed to the right at follow-up (Figure 7) but regarded as adequately normal distributed to fit the linear mixed model. The residuals were approximately normally distributed (Figure 8).

A linear mixed effects model showed that the yoga intervention seem to lead to moderate improvement in MAAS in the treatment group compared to the control group at post intervention (d=0·37, p=0·0063), but this intervention effect was considerably reduced and became non-significant at follow-up (table 4). There was a significant reduction in treatment effect between post-intervention and follow-up, estimated by the interaction between time and randomisation, (p=0·0060, table 4)


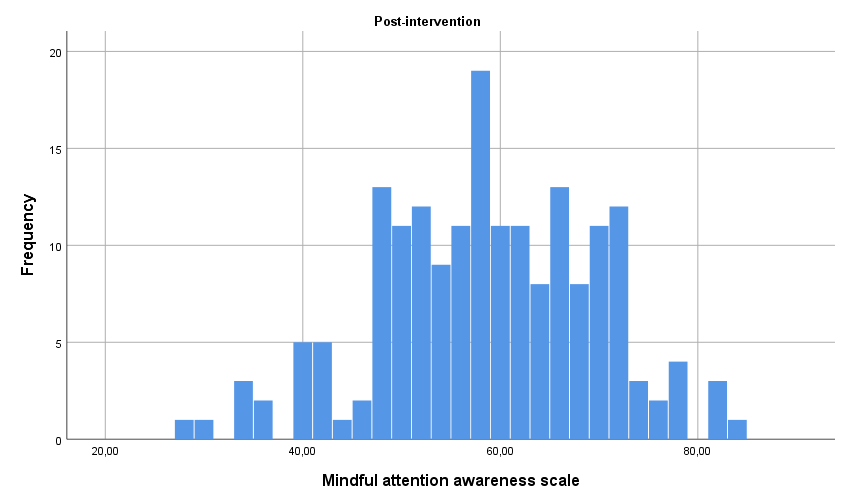


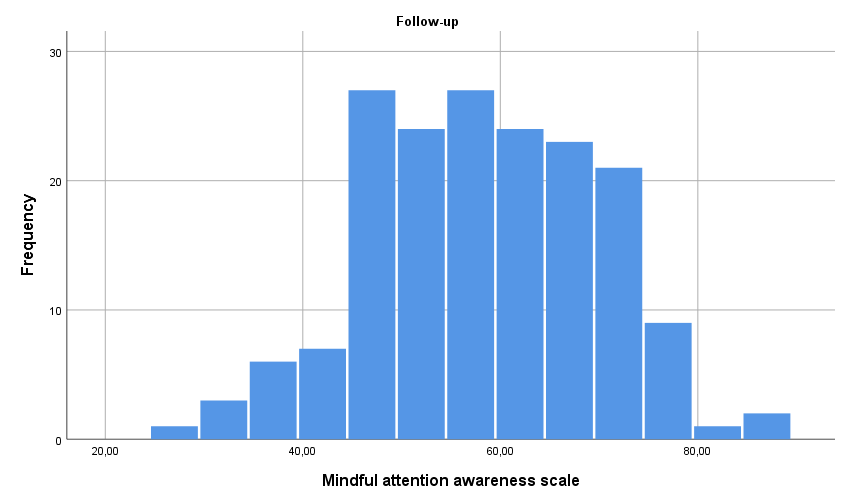


**FIGURE 7. HISTOGRAMS OF MAAS SCORES AT POST-INTERVENTION AND FOLLOW-UP**


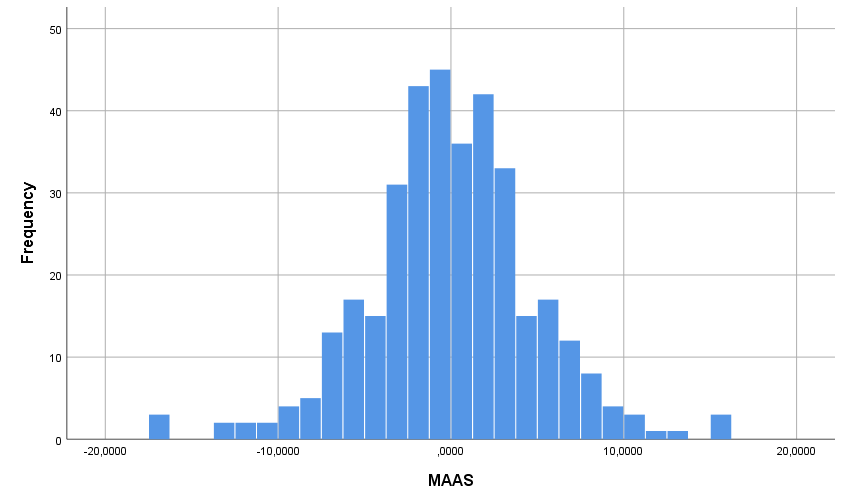


**FIGURE 8. HISTOGRAMS OF MAAS RESIDUALS.**

*Insomnia*

Insomnia was measured using the 6-item Bergen Insomnia Scale.^9^ The respondents are asked to rate how many days they experience six different problems with sleeping during a week, ranging from 0 days to 7 days. Scores can range from 0 to 42, high scores indicate more insomnia or problems with sleeping.

The distribution of the scores on BIS was found to be skewed to the right at both post-test and follow-up (Figure 9) but regarded as adequately normal distributed to fit the linear mixed model. The distribution of residuals was approximately normally distributed (Figure 10).

A linear mixed effects model showed that the yoga intervention seem to lead to moderate reduction in insomnia symptoms in the treatment group compared to the control group at both post-intervention (d= -0·41, p=0·0063), and at follow-up (d=-0·30, p=0·0121) (table 4). There was no significant difference in treatment effect between post-intervention and follow-up, estimated by the interaction between time and randomization, (p=0·3407, table 4)

**
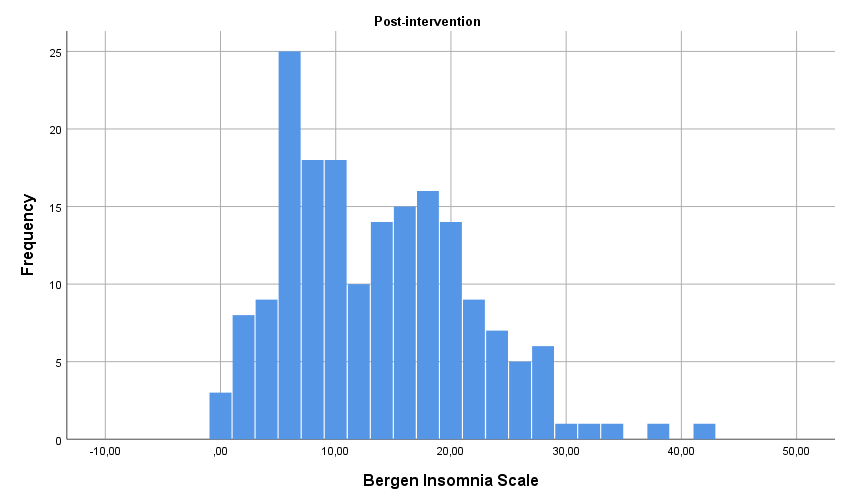
**

**
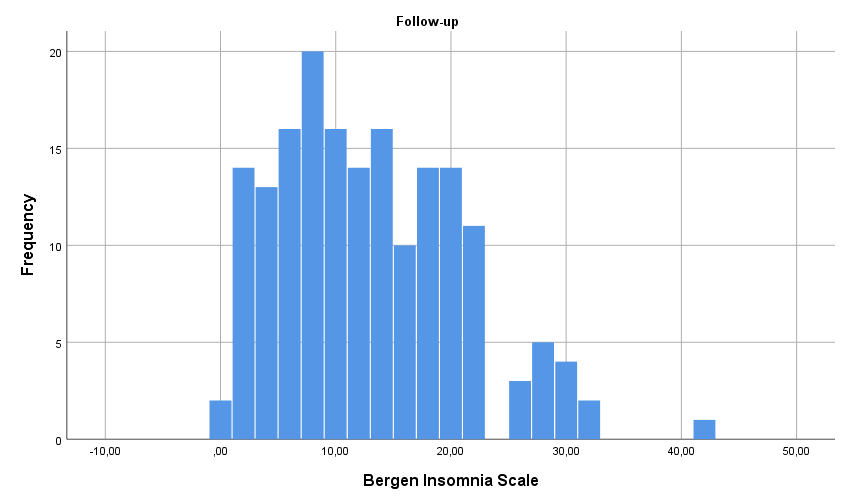
**

**FIGURE 9. HISTOGRAMS OF BIS SCORES AT POST-INTERVENTION AND FOLLOW-UP**


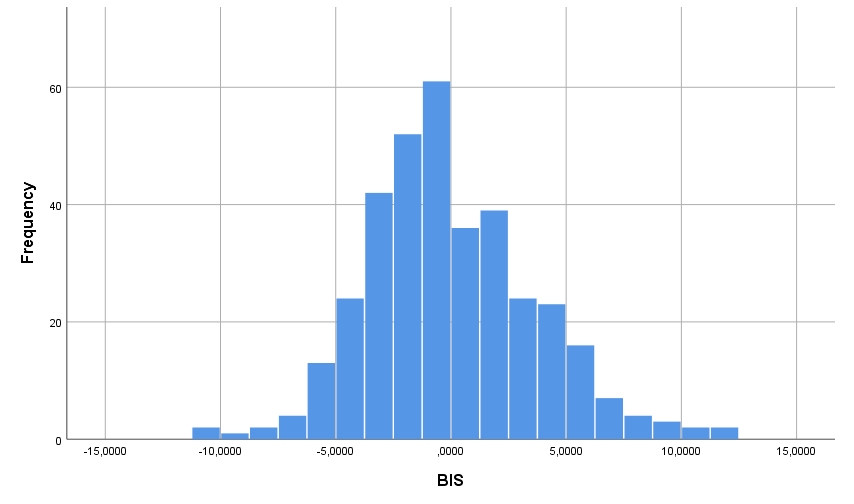


**FIGURE 10. HISTOGRAMS OF BIS RESIDUALS.**

**3.3.2 Secondary outcome HRV**

Heart rate variability was measured by The Root Mean Square of Successive Differences (RMSSD), which is the square root of the average of the squared differences between successive R-R intervals. The HRV data was recorded on Polar V800 pulse watches and belts overnight when the participants were sleeping, collecting nocturnal HRV. A time interval of 4 hours during night was set for the nocturnal data collection.

The distribution of the scores on the secondary outcome was found to be somewhat skewed to the right at post-test (Figure 11), but still regarded to be sufficiently normal distributed to fit the linear mixed effects model. Residuals were approximately normally distributed (Figure 12).


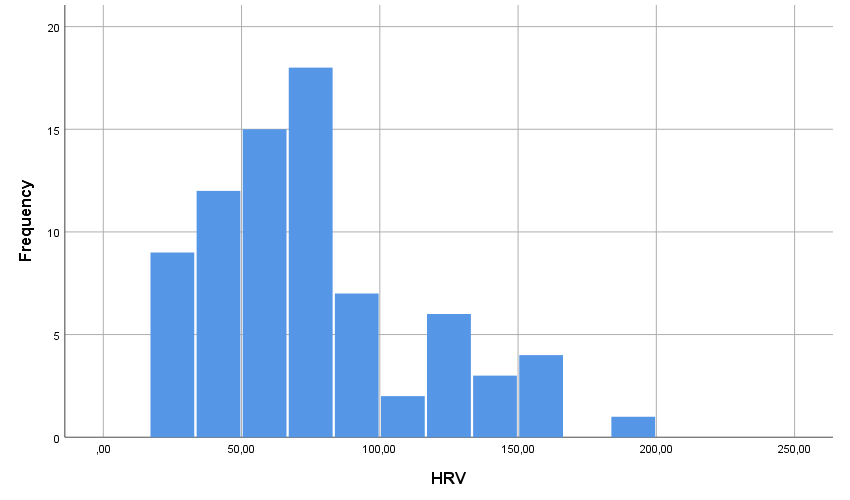


**FIGURE 11. HISTOGRAM OF HRV SCORES AT POSTTTEST**


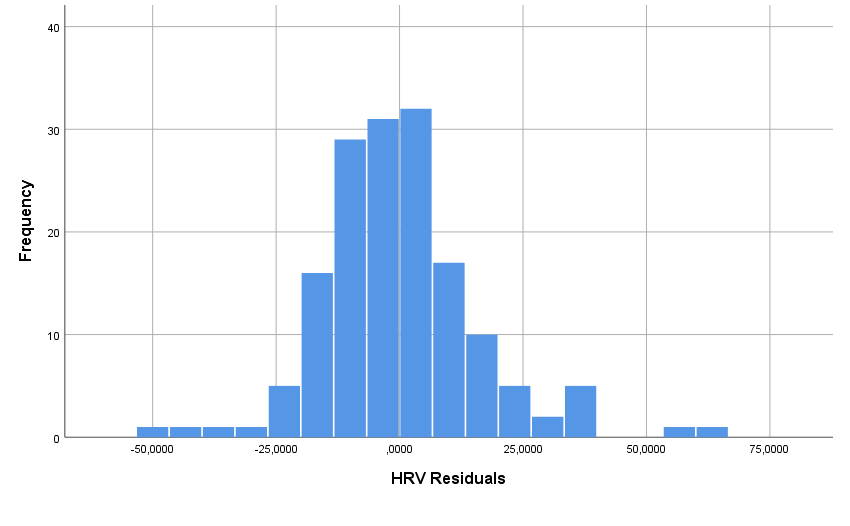


**FIGURE 12. HISTOGRAM OF HRV RESIDUALS.**

No significant difference (p = 0·2194) was found between the intervention group and the control group in the mean score on the secondary outcome HRV on posttest (table 5). The change in HRV-score from baseline to posttest was not significantly different between the intervention group and the control group, Group x Time, b = -2·24 (p = 0·7595).

**TABLE 5. SECONDARY OUTCOME RESULTS**

|  | **Heart rate variability (HRV)** | | | |
| --- | --- | --- | --- | --- |
|  | Unadjusted mean | | Difference (95% CI), d† | p value* |
|  | Control | Treatment |  |  |
| Post-intervention | 69·87 (34·70) | 80·60  (41·69) | 9·61 (- 5·80 to 25·02), 0·25 | 0·2194 |
| Data are mean (SD). At baseline, 37 participants were in the control group and 44 participants were in the treatment group. At post-intervention, 38 participants were in the control group and 39 participants were in the treatment group. HRV=Heart rate variability. *Linear mixed effects model with time (baseline vs post intervention), group and interaction of time with group, and including a random intercept for participants. Covariance matrix of within subject measurements was variance components. †d is standardised effect size (Cohen’s d). | | | | |

**3.4 Additional analyses using the baseline score as a timepoint**

Additional linear mixed effect models (LMM) for the primary and secondary outcomes were estimated using the score on each outcom at baseline as the first point in time, post-intervention as the second point in time and follow-up as the third point in time. This analysis estimates the change from baseline to post-intervention, and from baseline to follow-up. The model included one dummy-coded fixed effect for *Group* (control= 0, yoga=1), two dummy-coded fixed effects for time-point; *Time_1_* (post-intervention=1, else=0) and *Time_2_* (follow-up=1, else=0) and two interaction effects. The first interaction effect, *Time_1_×Group*, estimated whether the change from baseline to post-intervention was different for the two groups. The second interaction effect, *Time_2_×Group*, estimated whether the change from baseline to follow-up was different for the two groups. Thus, the interaction effect are estimates of effects of the intervention.

**3.4.1 Primary outcome**

The results from the LMM analysis presented in table 6 show that the effect of *Time_1_* and *Time* _2_ were non-significant. This means that for the control group, the scores on HSCL-25 did not change significantly from baseline to post-intervention and from baseline to follow-up. The two interaction terms between Time and group were statistically significant. The negative sign of the regression coefficients for both interaction terms suggested that mean scores on HSCL-25 was reduced from baseline to post-intervention and from baseline to follow-up in the yoga group only. On the basis of the parameters in the LMM model, the development over time in mean values on HSCL-25 is presented in figure 13.

**TABLE 6. ADDITIONAL ANALYSES OF PSYCHOLOGICAL DISTRESS USING BASELINE SCORE AS A TIMEPOINT**

|  | **Psychological distress (HSCL-25)** | | |
| --- | --- | --- | --- |
|  | b (SE) | 95% CI | p-value |
| *Intercept* (mean score for control group at baseline) | 1·84 (0·05) | (1·75 to 1·93) | < 0·0001 |
| *Group* (mean difference between control and yoga at baseline) | 0·07 (0·07) | (-0·06 to 0·20) | 0·2705 |
| *Time_1_* (overall mean change, baseline-postinterv.) | -0·01 (0·04) | (-0·10 to 0·07) | 0·7962 |
| *Time_2_* (overall mean change, baseline-follow-up) | -0·05 (0·04) | (-0·13 to 0·04) | 0·2623 |
| *Time_1_×Group* (mean change, baseline-postinterv. for yoga) | -0·18 (0·06) | (-0·30 to -0·06) | 0·0035 |
| *Time_2_×Group* (mean change, baseline-follow-up for yoga) | -0·21 (0·06) | (-0·33 to -0·09) | 0·0009 |
| Data are regression coefficients (SE). At baseline, 102 participants were in the control group, and 100 participants were in the yoga group. At post-intervention, 93 participants were in the control group and 89 participants were in the yoga group. At follow-up, 92 participants were in the control group and 83 participants were in the yoga group. HSCL-25=Hopkins Symptoms Checklist 25. *Linear mixed effects model using HSCL-25 as the dependent variable, time and interaction of time with group, and including a random intercept for participants. Group (control= 0, yoga=1), Time_1_ (post-intervention=1, else=0) and Time_2_ (follow-up=1, else=0). Covariance matrix of within subject measurements was variance components. | | | |


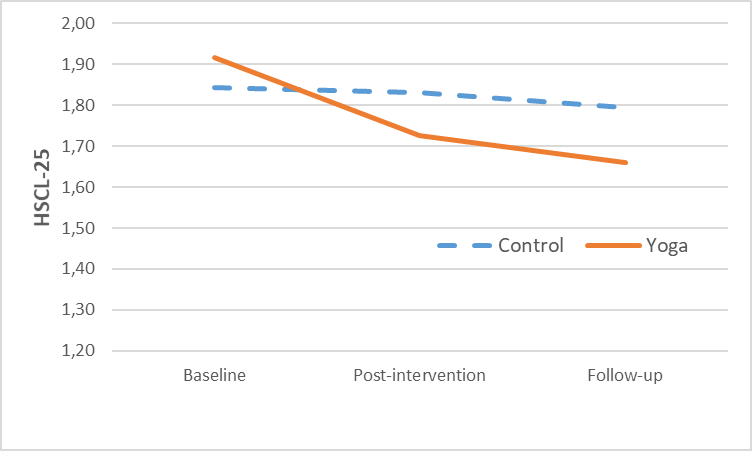


**Figure 13. The development in mean values on HSCL-25 over time, presented separately for the control and the yoga group. Values calculated from table 6.**

**3.4.2. Secondary outcomes**

**3.4.2.1 Satisfaction with life**

The results from the LMM analysis presented in table 7 show that the effect of Time1 and Time 2 were non-significant, i.e. for the control group, the scores on SWLS did not change significantly from baseline to post-intervention and from baseline to follow-up. The significant and positive sign of the regression coefficients for both interaction terms showed that mean scores on SWLS increased from baseline to post-intervention and from baseline to follow-up in the yoga group only. On the basis of the parameters in the LMM model, the development over time in mean values on SWLS is presented in figure 14.

**TABLE 7. ADDITIONAL ANALYSIS OF SATISFACTION WITH LIFE USING BASELINE SCORE AS A TIMEPOINT**

|  | **Satisfaction with life (SWLS)** | | |
| --- | --- | --- | --- |
|  | b (SE) | 95% CI | p-value |
| *Intercept* (mean score for control group at baseline) | 22·61 (0·60) | 21·44 to 23·78 | 0·0000 |
| *Group* (mean difference between control and yoga at baseline) | -1·14 (0·85) | -2·80 to 0·53 | 0·1798 |
| *Time_1_* (overall mean change, baseline-postinterv.) | -0·48 (0·50) | -1·45 to 0·50 | 0·3359 |
| *Time_2_* (overall mean change, baseline-follow-up) | 0·06 (0·50) | -0·92 to 1·04 | 0·9021 |
| *Time_1_×Group* (mean change, baseline-postinterv. for yoga) | 2·38 (0·70) | 1·00 to 3·76 | 0·0008 |
| *Time_2_×Group* (mean change, baseline-follow-up for yoga) | 1·57 (0·72) | 0·16 to 2·97 | 0·0295 |
| Data are regression coefficients (SE). At baseline, 102 participants were in the control group, and 100 participants were in the yoga group. At post-intervention, 93 participants were in the control group and 89 participants were in the yoga group. At follow-up, 92 participants were in the control group and 83 participants were in the yoga group. Linear mixed effects model using SWLS as the dependent variable, time and interaction of time with group, and including a random intercept for participants. Group (control= 0, yoga=1), Time_1_ (post-intervention=1, else=0) and Time_2_ (follow-up=1, else=0). Covariance matrix of within subject measurements was variance components. | | | |


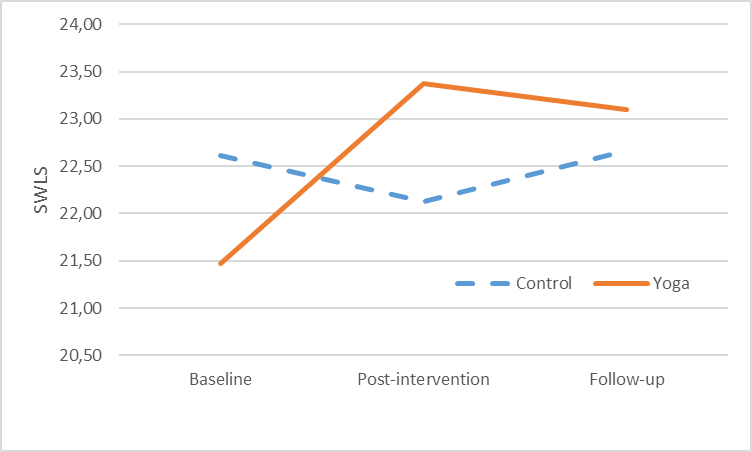


**FIGURE 14. THE DEVELOPMENT IN MEAN VALUES ON SWLS OVER TIME, PRESENTED SEPARATELY FOR THE CONTROL AND THE YOGA GROUP. VALUES CALCULATED FROM TABLE 7.**

**3.4.2.2 Warwick-Edinburg Mental Well-Being Scale (WEMWBS).**

The results from the LMM analysis presented in table 8 show that the effect of Time1 and Time 2 were non-significant, i.e. for the control group, the scores on WEMWBS did not change significantly from baseline to post-intervention and from baseline to follow-up. The significant and positive sign of the regression coefficients for *Time_1_×Group* showed that mean score on WEMWBS increased from baseline to post-intervention within the yoga group. However, no significant increase in WEMWBS mean scores was found from baseline to follow-up in the yoga group. On the basis of the parameters in the LMM model, the development over time in mean values on WEMWBS is presented in figure 16.

**TABLE 8. ADDITIONAL ANALYSES OF WARWICK-EDINBURG MENTAL WELL-BEING SCALE USING BASELINE SCORE AS A TIMEPOINT**

| **Warwick-Edinburg Mental Well-Being Scale (WEMWBS)** | | | |
| --- | --- | --- | --- |
|  | b (SE) | 95% CI | p-value |
| *Intercept* (mean score for control group at baseline) | 46·15 (0·85) | 44·47 to 47·82 | 0·0000 |
| *Group* (mean difference between control and yoga at baseline) | -0·88 (1·21) | -3·26 to 1·50 | 0·4691 |
| *Time_1_* (overall mean change, baseline-postinterv.) | -0·67 (0·81) | -2·25 to 0·91 | 0·4054 |
| *Time_2_* (overall mean change, baseline-follow-up) | 0·79 (0·81) | -0·80 to 2·38 | 0·3279 |
| *Time_1_×Group* (mean change, baseline-postinterv. for yoga) | 3·07 (1·14) | 0·82 to 5·31 | 0·0076 |
| *Time_2_×Group* (mean change, baseline-follow-up for yoga) | 0·85 (1·16) | -1·44 to 3·13 | 0·4671 |
| Data are regression coefficients (SE). At baseline, 102 participants were in the control group, and 100 participants were in the yoga group. At post-intervention, 93 participants were in the control group and 89 participants were in the yoga group. At follow-up, 92 participants were in the control group and 83 participants were in the yoga group. Linear mixed effects model using WEMWBS as the dependent variable, time and interaction of time with group, and including a random intercept for participants. Group (control= 0, yoga=1), Time_1_ (post-intervention=1, else=0) and Time_2_ (follow-up=1, else=0). Covariance matrix of within subject measurements was variance components. | | | |


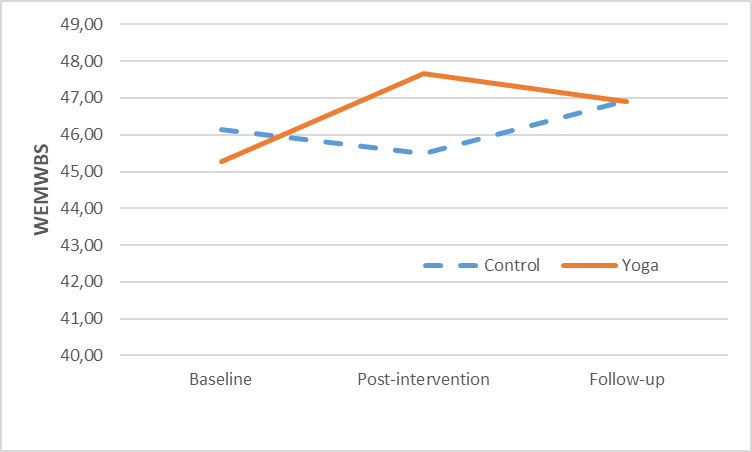


**FIGURE 15. THE DEVELOPMENT IN MEAN VALUES ON WARWICK-EDINBURG MENTAL WELL-BEING SCALE OVER TIME, PRESENTED SEPARATELY FOR THE CONTROL AND THE YOGA GROUP. VALUES CALCULATED FROM TABLE 8.**

**3.4.2.3 Mindful Attention Awareness Scale (MAAS)**

The results from the LMM analysis presented in table 9 show that the effect of *Time_1_* was non-significant, i.e. for the control group, the scores on MAAS did not change significantly from baseline to post-intervention. However, the coefficient for *Time_1_* was significant, suggesting an increase in mindfulness from baseline to follow-up in the control group. The significant and positive sign of the regression coefficients for *Time_1_×Group* showed that mean score on MAAS increased from baseline to post-intervention within the yoga group. However, no significant increase in MAAS mean scores was found from baseline to follow-up in the yoga group. On the basis of the parameters in the LMM model, the development over time in mean values on WEMWBS is presented in figure 16.

**TABLE 9. ADDITIONAL ANALYSES OF MINDFUL ATTENTION AWARENESS SCALE USING BASELINE SCORE AS A TIMEPOINT**

| **Mindful Attention Awareness Scale (MAAS)** | | | |
| --- | --- | --- | --- |
|  | b (SE) | 95% CI | p-value |
| *Intercept* (mean score for control group at baseline) | 55·12 (1·14) | 52·88 to 57·35 | 0·0000 |
| *Group* (mean difference between control and yoga at baseline) | -1·70 (1·62) | -4·88 to 1·48 | 0·2940 |
| *Time_1_* (overall mean change, baseline-postinterv.) | 1·27 (1·03) | -0·77 to 3·30 | 0·2214 |
| *Time_2_* (overall mean change, baseline-follow-up) | 3·03 (1·04) | 0·98 to 5·07 | 0·0038 |
| *Time_1_×Group* (mean change, baseline-postinterv. for yoga) | 5·15 (1·47) | 2·27 to 8·04 | 0·0005 |
| *Time_2_×Group* (mean change, baseline-follow-up for yoga) | 1·51 (1·49) | -1·43 to 4·45 | 0·3126 |
| Data are regression coefficients (SE). At baseline, 102 participants were in the control group, and 100 participants were in the yoga group. At post-intervention, 93 participants were in the control group and 89 participants were in the yoga group. At follow-up, 92 participants were in the control group and 83 participants were in the yoga group. Linear mixed effects model using MAAS as the dependent variable, time and interaction of time with group, and including a random intercept for participants. Group (control= 0, yoga=1), Time_1_ (post-intervention=1, else=0) and Time_2_ (follow-up=1, else=0). Covariance matrix of within subject measurements was variance components. | | | |


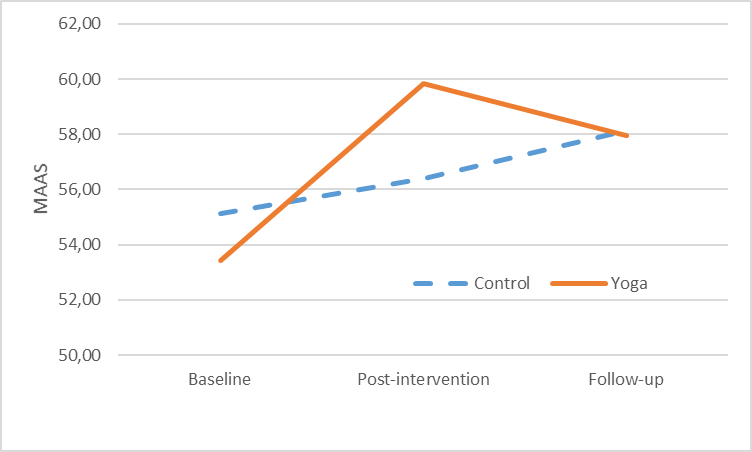


**FIGURE 16. THE DEVELOPMENT IN MEAN VALUES ON MINDFUL ATTENTION AWARENESS SCALE OVER TIME, PRESENTED SEPARATELY FOR THE CONTROL AND THE YOGA GROUP. VALUES CALCULATED FROM TABLE 9.**

**3.4.2.4 Bergen Insomnia Scale (BIS)**

The results from the LMM analysis presented in table 10 show that the effect of *Time_1_* and *Time* _2_ were non-significant. This means that for the control group, the scores on BIS did not change significantly from baseline to post-intervention and from baseline to follow-up. The two interaction terms between Time and group were statistically significant. The negative sign of the regression coefficients for both interaction terms suggested that mean scores on BIS was reduced from baseline to post-intervention and from baseline to follow-up in the yoga group only. On the basis of the parameters in the LMM model, the development over time in mean values on BIS is presented in figure 17.

**TABLE 10. ADDITIONAL ANALYSES OF BERGEN INSOMNIA SCALE USING BASELINE SCORE AS A TIMEPOINT**

| **Bergen Insomnia Scale (BIS)** | | | |
| --- | --- | --- | --- |
|  | b (SE) | 95% CI | p-value |
| *Intercept* (mean score for control group at baseline) | 14·30 (0·80) | 12·73 to 15·88 | 0·0000 |
| *Group* (mean difference between control and yoga at baseline) | 1·70 (1·14) | -0·54 to 3·93 | 0·1370 |
| *Time_1_* (overall mean change, baseline-postinterv.) | 0·21 (0·77 | -1·30 to 1·72 | 0·7819 |
| *Time_2_* (overall mean change, baseline-follow-up) | -0·80 (0·77) | -2·31 to 0·72 | 0·3026 |
| *Time_1_×Group* (mean change, baseline-postinterv. for yoga) | -4·25 (1·09) | -6·39 to -2·11 | 0·0001 |
| *Time_2_×Group* (mean change, baseline-follow-up for yoga) | -3·36 (1·11) | -5·54 to -1·17 | 0·0027 |
| Data are regression coefficients (SE). At baseline, 102 participants were in the control group, and 100 participants were in the yoga group. At post-intervention, 93 participants were in the control group and 89 participants were in the yoga group. At follow-up, 92 participants were in the control group and 83 participants were in the yoga group. Linear mixed effects model using BIS as the dependent variable, time and interaction of time with group, and including a random intercept for participants. Group (control= 0, yoga=1), Time_1_ (post-intervention=1, else=0) and Time_2_ (follow-up=1, else=0). Covariance matrix of within subject measurements was variance components. | | | |


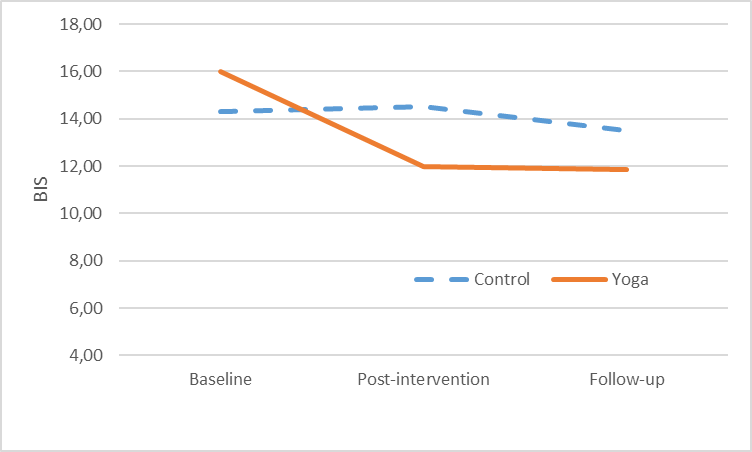


**FIGURE 17. THE DEVELOPMENT IN MEAN VALUES ON BERGEN INSOMNIA SCALE OVER TIME, PRESENTED SEPARATELY FOR THE CONTROL AND THE YOGA GROUP. VALUES CALCULATED FROM TABLE 10.**

**3.5 Missing data mechanism**

**3.5.1 Primary outcome**

The intention to treat (ITT) population consisted of 202 participant who were randomized to either the yoga group of the control group. Differences between the groups on the primary outcome, HSCL-25, was analyzed assuming data were missing at random (MAR).

Drop-out, referring to missingness on the primary outcome, was regarded as low as only 17 of the 202 participants had missing values at both post-intervention and follow-up. Table 11 shows that there was no significant association between drop-out/missingness and the group the participants were assigned to. Furthermore, missingness was not related to characteristics such as age, gender and HSCL-25 score at baseline. The p-values in table 11 refers the result from bivariate logistic regression models using baseline characteristics to predicting missingness.

**TABLE 11. BASELINE CHARACTERISTICS PREDICTING MISSINGNESS AT POST-TEST AND FOLLOW-UP ON THE PRIMARY OUTCOME HSCL-25 AND THE SECONDARY OUTCOMES SWLS, WEMWBS, MAAS, AND BIS. LOGISTIC REGRESSION MODELS.**

| Baseline charcteristics | **Predicting missingness** | **Control group** |  | **Intervention group** |  |
| --- | --- | --- | --- | --- | --- |
|  | *Study arm p = 0·7672* | *Missing (N= 8)* | *Not missing (N=94)* | *Missing (N=9)* | *Not missing (N=91)* |
| Age | p = 0·7338 | 25·25 (2·44) | 24·98 (4·16) | 24·78 (6·89) | 25·71 (3·78) |
| Sex | p = 0·5558 |  |  |  |  |
| Male |  | 2 (25·0%) | 10 (10·6%) | 1 (11·1%) | 13 (14·3%) |
| Female |  | 6 (75·0%) | 84 (89·4%) | 8 (88·9%) | 78 (85·7%) |
|  |  |  |  |  |  |
| Primary outcome at baseline |  |  |  |  |  |
| HSCL-25 | p = 0·8053 | 2·06 (0·59) | 1·82 (0·43) | 1·77 (0·53) | 1·93 (0·50) |
|  |  |  |  |  |  |
| HSCL-25 Cut-off | p = 0·8357 |  |  |  |  |
| Above (>1.75) |  | 6 (75%) | 48 (51·1%) | 56 (61·5%) | 4 (44·4%) |
| Below (≤ 1.75) |  | 2 (25%) | 46 (48·9 %) | 35 (38·5%) | 5 (55·5%) |
|  |  |  |  |  |  |
| Secondary outcomes at baseline |  |  |  |  |  |
| SWLS | p = 0·5601 | 20·25 (6·14) | 22·81 (5·90) | 22·11 (7·03) | 21·41 (5·98) |
| WEMWBS | p = 0·8727 | 45·13 (7·74) | 46·23 (8·25) | 45·67 (11·80) | 45·23 (7·70) |
| MAAS | p = 0·8582 | 52·63 (15·19) | 55·33 (11·63) | 56·67 (11·91) | 53·10 (11·68) |
| BIS | p = 0·6859 | 18·13 (7·08) | 13·98 (8·43) | 14·00 (5·83) | 16·20 (8·83) |
|  |  |  |  |  |  |

**3.5.2 Secondary outcomes SWLS, WEMWBS, MAAS and BIS**

The same 17 participants who had missing values on participants had missing values on the primary outcome had also missing values on the secondary outcomes SWLS, WEMWBS, MAAS and BIS at both post-intervention and follow-up. Differences between the two groups on the secondary outcomes SWLS, WEMWBS, MAAS and BIS, was analyzed assuming data were missing at random (MAR). Baseline scores on the secondary outcomes SWLS, WEMWBS, MAAS and BIS were not related to missingness (table 11).

**3.5.3 Secondary outcome HRV**

The intention to treat (ITT) population of the secondary outcome Heart Rate Variability consisted of 105 participant who were randomized to either the yoga group or the control group. Differences between the two study arms on the secondary outcome, HRV, was analyzed assuming data were missing at random (MAR). As shown in table 12, there was a significant relationship between age and misssingness; participants who dropped out or had invalid HRV measurement were on average 1.6 years older compared to participants with valid HRV-measurement.

**TABLE 12. BASELINE CHARACTERISTICS PREDICTING MISSINGNESS ON HRV AT POSTTEST. LOGISTIC REGRESSION MODELS. to**

| Baseline charcteristics | **Predicting missingness** | **Control group** |  | **Intervention group** |  |
| --- | --- | --- | --- | --- | --- |
|  | *Study arm p = 0·0753* | *Missing (N=15)* | *Not missing (N=38)* | *Missing (N=13)* | *Not missing (N=39)* |
| Age | p = 0·0264 | 25·27 (2·25) | 24·37 (2·85) | 27·00 (4·42) | 24·59 (2·98) |
| Sex | p = 0·0915 |  |  |  |  |
| Male |  | 3 (20·0%) | 1 (2·6%) | 2 (15·6%) | 4 (14·3%) |
| Female |  | 12 (80·0%) | 37 (97·4%) | 11 (84·4%) | 35 (89·7%) |
|  |  |  |  |  |  |
| Secondary outcome at baseline |  | *(N=7)* | *(N=30)* | *(N=8)* | *(N=36)* |
| HRV | p = 0·5213 | 72·29 (20·51) | 63·95 (30·84) | 55·75 (0·53) | 76·06 (37·15) |

**3.6 SENSITIVITY ANALYSIS**

**3.6.1 Primary outcome**

Table 13 shows that the treatment differences were consistent with the primary analyses in three types of sensitivity analyses. Per-protocol analysis, i.e. including only subjects having valid scores on both posttest and follow-up, gave significant treatment differences in favor of the intervention group. Restricting the subjects in the intervention group to those having 80% compliance or more gave only trivial changes in treatment differences compared to the primary analysis. Intention-to-treat analysis, i.e. the most conservative estimate, using multiple imputation of missing values on HSCL-25 also gave significant treatment differences.  **TABLE 13. SENSITIVITY ANALYSES OF THE PRIMARY OUTCOME**

|  | **Psychological distress (HSCL-25)** | | | |
| --- | --- | --- | --- | --- |
|  | Unadjusted mean | | Adjusted difference* (95% CI), d† | p value* |
|  | Control | Treatment |  |  |
| **Per-protocol analysis. Based on subjects with valid scores on all timepoints (listwise deletion)^a^** | | | | |
| Posttest | 1·81 (0·45) | 1·71 (0·43) | -0·15 (-0·26 to -0·03), -0·32 | 0·0124 |
|  |  |  |  |  |
| Follow-up | 1·78 (0·52) | 1·65 (0·45) | -0·17 (-0·29 to -0·06), -0·37 | 0·0034 |
| **Cases with 80 % compliance in the treatment condition (participated in 20 yoga sessions or more)^b^** | | | | |
| Posttest | 1·82 (0·46) | 1·71 (0·39) | -0·17 (-0·28 to -0·06), -0·36 | 0·0036 |
|  |  |  |  |  |
| Follow-up | 1·78 (0·52) | 1·66 (0·44) | -0·17 (-0·28 to -0·06), -0·36 | 0·0039 |
| **Intention-to-treat analysis. Multiple imputation of missing data^c^** | | | | |
| Posttest | 1·82 (0·46) | 1·74 (0·45) | -0·13 (-0·24 to -0·02), -0·28 | 0·0219 |
|  |  |  |  |  |
| Follow-up | 1·79 (0·52) | 1·68 (0·45) | -0·15 (-0·28 to -0·03), -0·32 | 0·0179 |
|  |  |  |  |  |
| Data are mean (SD). *Linear mixed effects model adjusted for baseline score on HSCL-25, time and interaction of time with randomisation, and including a random intercept for participants. HSCL-25=Hopkins Symptoms Checklist 25. Covariance matrix of within subject measurements was variance components. †d is standardised effect size (Cohen’s d).  ^a^ 91 participants were in the control group and 82 participants were in the treatment group.  ^b^At posttest, 93 participants were in the control group and 77 participants were in the treatment group. At follow-up, 92 participants were in the control group and 75 participants were in the treatment group  ^c^After randomization, 102 participants were in the control group and 100 participants were in the treatment group. Missing values imputed on HSCL-25. | | | | |

Table 14 shows sensitivity analyses based upon a cut-off value above 1·75 points in HSCL-25. In accordance with the primary analysis, the proportion scoring over the cut-off value of 1·75 was lower in the intervention group, but no significantly lower. **TABLE 14: SENSITIVITY ANALYSES OF PRIMARY OUTCOME USING >1.75 AS CUT OFF VALUE**

|  | **Psychological distress (HSCL-25)** | | | | |
| --- | --- | --- | --- | --- | --- |
|  | Above cut-off (1.75) | | Adjusted odds ratio^a^ (95% CI) | Adjusted risk ratio^b^ (95% CI) | p value^a^ |
|  | Control | Treatment |  |  |  |
| **Per-protocol analysis. Based on subjects with valid scores on all timepoints (listwise deletion)^c^** | | | | | |
| Post-intervention | 45 (49·5%) | 30 (37·0%) | 0·42 (0·21 to 0·85) | 0·59 (0·34 to 0·92) | 0·0156 |
| Follow-up | 38 (41·8%) | 28 (34·6%) | 0·57 (0·29 to 1·14) | 0·70 (0·41 to 1·08) | 0·1102 |
| **Cases with 80 % compliance in the treatment condition (participated in 20 yoga sessions or more)^d^** | | | | | |
| Post-intervention | 47 (50·5%) | 29 (38·2%) | 0·41 (0·20 to 0·84) | 0·58 (0·34 to 0·91) | 0·0144 |
| Follow-up | 39 (42·4%) | 25 (33·8%) | 0·54 (0·27 to 1·09) | 0·67 (0·39 to 1·05) | 0·0858 |
| **Intention-to-treat analysis. Multiple imputation of missing data^e^** | | | | | |
| Post-intervention | 52·8 (51·8%) | 40·2 (40·2%) | 0·46 (0·23 to 0·93) | 0·59 (0·38 to 0·97) | 0·0309 |
| Follow-up | 45·6 to (44·7%) | 37 (37·0%) | 0·58 (0·30 to 1·15) | 0·72 (0·43 to 1·08) | 0·1211 |
| Data are N (%). At post-intervention, 93 participants were in the control group and 89 participants were in the treatment group. At follow-up, 92 participants were in the control group and 83 participants were in the treatment group. HSCL-25=Hopkins Symptoms Checklist 25. ^a^Logistic regression model using adjusted for number of participants within each group scoring under or above cut-off score on HSCL-25. ^b^Risk ratio estimated from adjusted odds ratio. to ^c^91 participants were in the control group and 81 participants were in the treatment group. to ^d^At posttest, 93 participants were in the control group and 76 participants were in the treatment group. At follow-up, 92 participants were in the control group and 74 participants were in the treatment group to ^e^After randomization, 102 participants were in the control group and 100 participants were in the treatment group. Missing values imputed on HSCL-25. | | | | | |

**3.6.2 Secondary outcomes**

Table 15 shows that the treatment differences were consistent with the primary analyses in three types of sensitivity analyses for SWLS, WEMWBS, MAAS and BIS. Per-protocol analysis, i.e. including only subjects having valid scores on both posttest and follow-up, gave significant treatment differences in favor of the intervention group at post-intervention but not at follow-up for SWLS, WEMWBS, MAAS. Per-protocol analysis for BIS gave significant treatment differences in favor of the intervention group at both post-intervention and follow-up, in accordance with the primary analyses.

Restricting the subjects in the intervention group to those having 80% compliance or more gave only trivial changes in treatment differences compared to the primary analysis of SWLS, WEMWBS, MAAS and BIS.

Intention-to-treat analysis, i.e. the most conservative estimate, using multiple imputation of missing values on SWLS, WEMWBS, MAAS and BIS also gave significant treatment differences at post-intervention for all secondary outcomes. At follow-up only a significant treatment effect for BIS was found. To sum up, all three types of sensitivity analyses gave the same results as the primary analyses **TABLE 15. SENSITIVITY ANALYSES OF THE SECONDARY OUTCOMES SWLS, WEMWBS, MAAS AND BIS**

|  | Unadjusted mean | | Adjusted difference* (95% CI), d† | p value* |
| --- | --- | --- | --- | --- |
|  | Control | Treatment |  |  |
| **Per-protocol analysis. Based on subjects with valid scores on all timepoints (listwise deletion)^a^** | | | | |
| SWLS |  |  |  |  |
| Post-intervention | 22·30 (6·18) | 23·81 (5·29) | 2·33 (0·99 to 3·68), 0·39 | 0·0007 |
| Follow-up | 22·87 (6·78) | 23·35 (5·90) | 1·29 (-0·06 to 2·64), 0·22 | 0·0602 |
| WEMWBS |  |  |  |  |
| Post-intervention | 45·74 (8·80) | 48·41 (7·32) | 3·00 (0·82 to 5·18), 0·39 | 0·0073 |
| Follow-up | 47·11 (9·88) | 47·44 (8·85) | 0·66 (-1·52 to 2·85), 0·09 | 0·5510 |
| MAAS |  |  |  |  |
| Post-intervention | 56·69 (11·01) | 59·94 (10·39) | 4·05 (1·28 to 6·83), 0·36 | 0·0043 |
| Follow-up | 58·43 (11·79) | 58·35 (11·50) | 0·72 (-2·05 to 3·50), 0·06 | 0·6075 |
| BIS |  |  |  |  |
| Post-intervention | 14·15 (8·24) | 11·40 (7·25) | -4·07 (-6·04 to -2·10), -0·47 | 0·0001 |
| Follow-up | 13·22 (8·84) | 11·65 (6·87) | -2·87 (-4·85 to -0·90), -0·33 | 0·0044 |
| **Cases with 80 % compliance in the treatment condition (participated in 20 yoga sessions or more)^b^** | | | | |
| SWLS |  |  |  |  |
| Post-intervention | 22·27 (6·13) | 23·70 (5·27) | 2·83 (0·83 to 3·53), 0·48 | 0·0016 |
| Follow-up | 22·86 (6·75) | 23·52 (5·95) | 1·33 (-0·03 to 2·61), 0·22 | 0·0547 |
| WEMWBS |  |  |  |  |
| Post-intervention | 45·52 (8·87) | 48·00 (7·54) | 2·90 (0·78 to 5·03), 0·35 | 0·0075 |
| Follow-up | 47·15 (9·84) | 47·76 (8·10) | 0·87 (-1·27 to 3·01), 0·11 | 0·4223 |
| MAAS |  |  |  |  |
| Post-intervention | 56·59 (10·95) | 59·35 (10·51) | 3·92 (1·12 to 6·72), 0·37 | 0·0062 |
| Follow-up | 58·32 (11·37) | 57·91 (11·48) | 0·76 (-2·05 to 3·58), 0·07 | 0·5933 |
| BIS |  |  |  |  |
| Post-intervention | 14·34 (8·38) | 11·26 (6·86) | -4·12 (-6·13 to -2·10), -0·47 | 0·0005 |
| Follow-up | 13·17 (8·59) | 11·65 (6·70) | -2·63 (-4·67 to -0·60), -0·24 | 0·0113 |
| **Intention-to-treat analysis. Multiple imputation of missing data^c^** | | | | |
| SWLS |  |  |  |  |
| Post-intervention | 22·24 (6·13) | 22·78 (5·37) | 1·78 (0·52 to 3·04), 0·31 | 0·0058 |
| Follow-up | 23·25 (6·62) | 22·98 (5·88) | 0·97 (-0·55 to 2·49), 0·17 | 0·2058 |
| WEMWBS |  |  |  |  |
| Post-intervention | 45·66 (8·80) | 47·64 (7·75) | 2·55 (0·88 to 4·22), 0·31 | 0·0030 |
| Follow-up | 46·94 (9·85) | 47·06 (8·79) | 0·63 (-1·04 to 2·30), 0·08 | 0·4596 |
| MAAS |  |  |  |  |
| Post-intervention | 56·63 (10·91) | 59·91 (10·49) | 4·19 (1·96 to 6·41), 0·39 | 0·0003 |
| Follow-up | 57·72 (11·89) | 58·14 (11·58) | 1·29 (-0·94 to 3·52), 0·12 | 0·2558 |
| BIS |  |  |  |  |
| Post-intervention | 14·23 (8·26) | 12·18 (7·31) | -2·92 (-4·48 to -1·37), -0·37 | 0·0003 |
| Follow-up | 13·33 (8·60) | 11·80 (7·01) | -2·33 (-3·88 to -0·77), -0·30 | 0·0036 |
| Data are mean (SD). At post-intervention, 93 participants were in the control group and 89 participants were in the treatment group. At follow-up, 92 participants were in the control group and 83 participants were in the treatment group. SWLS= Satisfaction With Life Scale, WEMWMS= Warwick-Edinburgh Mental Wellbeing Scale, MAAS = Mindful Attention Awareness Scale, BIS=Bergen Insomnia Scale. *Linear mixed effects model adjusted for baseline score, time and interaction of time with randomisation, and including a random intercept for participants. Covariance matrix of within subject measurements was variance components. †d is standardised effect size (Cohen’s d). to ^a^ 91 participants were in the control group and 82 participants were in the treatment group. to ^b^At posttest, 93 participants were in the control group and 77 participants were in the treatment group. At follow-up, 92 participants were in the control group and 75 participants were in the treatment group to ^c^After randomization, 102 participants were in the control group and 100 participants were in the treatment group. Missing values imputed. | | | | |

Table 16 shows that the treatment differences were consistent with the secondary analyses of HRV in three types of sensitivity analyses, meaning that no significant differences in HRV were found between the treatment group and the control group.

**TABLE 16: SENSITIVITY ANALYSES OF SECONDARY OUTCOME HRV**

|  | **Heart rate variability (HRV)** | | | |
| --- | --- | --- | --- | --- |
|  | Unadjusted mean | | Adjusted difference* (95% CI), d† | p value* |
|  | Control | Treatment |  |  |
| **Per-protocol analysis. Based on subjects with valid scores on all timepoints (listwise deletion)^a^** | | | | |
| Posttest | 69·40 (35·40) | 78·31 (41·33) | 14·06 (- 4·13 to 32·24), 0·34 | 0·1283 |
|  |  |  |  |  |
| **Cases with 80 % compliance in the treatment condition (participated in 20 yoga sessions or more)^b^** | | | | |
| Posttest | 69·87 (34·70) | 76·54 (38·79) | 9·08 (-6·01 to 24·14), 0·25 | 0·2359 |
|  |  |  |  |  |
| **Intention-to-treat analysis. Multiple imputation of missing data^c^** | | | | |
| Posttest | 71·21 (34·70) | 77·58 (41·69) | 8·67 (- 9·36 to 26·69), 0·23 | 0·3346 |
|  |  |  |  |  |
| Data are mean (SD). HRV=Heart rate variability. *Linear mixed effects model with time (baseline vs post intervention), group and interaction of time with group, and including a random intercept for participants. Covariance matrix of within subject measurements was variance components. †d is standardised effect size (Cohen’s d). to ^a^ 30 participants were in the control group and 36 participants were in the treatment group. to ^b^At posttest, 37 participants were in the control group and 42 participants were in the treatment group ^to c^ After randomization, 53 participants were in the control group and 52 participants were in the treatment group. Missing values imputed on the basis of age, sex and baseline score on HRV or valid posttest score on HRV (for participants having missing HRV-value at baseline). | | | | |

**3.7 Association between HRV and primary outcome and secondary outcomes**

Pearsons product-moment correlations revealed no significant associations between heart rate variability and psychological distress, sleep or well-being at either baseline or post-test (Table 17 and Table 18).

**TABLE 17: ASSOCIATIONS BETWEEN HRV AND PRIMARY AND SECONDARY OUTCOMES AT BASELINE. ZERO-ORDER CORRELATIONS**

| Baseline characteristics | **HRV-score baseline*** |  |
| --- | --- | --- |
| HSCL-25 | -0·160 | p = 0·1537 |
| SWLS | 0·053 | p = 0·6366 |
| WEMWBS | 0·211 | p = 0·0590 |
| MAAS | -0·097 | p = 0·3889 |
| BIS | -0·106 | p = 0·3470 |
|  |  |  |
| HRV= Heart rate variability, HSCL-25=Hopkins Symptoms Checklist 25, SWLS= Satisfaction With Life Scale, WEMWMS= Warwick-Edinburgh Mental Wellbeing Scale, MAAS = Mindful Attention Awareness Scale, BIS=Bergen Insomnia Scale. N = 81. *Pearsons product –moment correlation | | |

**TABLE 18: ASSOCIATIONS BETWEEN HRV AND PRIMARY AND SECONDARY OUTCOMES AT POSTTEST. ZERO-ORDER CORRELATIONS**

| Posttest characteristics | **HRV- score posttest** |  |
| --- | --- | --- |
| HSCL-25 | -0·125 | p = 0·2792 |
| SWLS | 0·038 | p = 0·7440 |
| WEMWBS | 0·071 | p = 0·5390 |
| MAAS | 0·109 | p = 0·3447 |
| BIS | 0·148 | p = 0·2002 |
|  |  |  |
| HRV= Heart rate variability, HSCL-25=Hopkins Symptoms Checklist 25, SWLS= Satisfaction With Life Scale, WEMWMS= Warwick-Edinburgh Mental Wellbeing Scale, MAAS = Mindful Attention Awareness Scale, BIS=Bergen Insomnia Scale. N = 77. *Pearsons product –moment correlation | | |

**4. REFERENCES**

1. Tambs K, Røysamb E. Selection of questions to short-form versions of original psychometric instruments in MoBa. *Norsk Epidemiologi* 2014; **24**: 195-201.

2. Strand BH, Dalgard OS, Tambs K, Rognerud M. Measuring the mental health status of the Norwegian population: a comparison of the instruments SCL-25, SCL-10, SCL-5 and MHI-5 (SF-36). *Nordic journal of psychiatry* 2003; **57**(2): 113-8.

3. Williams JW, Noël PH, Cordes JA, et al. Is This Patient Clinically Depressed? *JAMA* 2002; **287**(9): 1160-1170. Doi: 10.1001/jama.287.9.1160

4. Johansen R, Rognerud M, Sundet JM. Levekårsundersøkelsene 1998, 2002 og 2005: Utvikling i psykisk helse. *Nasjonalt folkehelseinstitutt.* 2008; **4**: 1-41.

5. Sivertsen B, Råkil H, Munkvik E, Lønning KJ. Cohort profile: the SHoT-study, a national health and well-being survey of Norwegian university students. *BMJ Open* 2019; **9**(1): e025200.

6. Pavot W, Diener E. The Satisfaction With Life Scale and the emerging construct of life

satisfaction. The Journal of Positive Psychology. 2008;**3**(2):137-52.

7. Smith ORF, Alves DE, Knapstad M, Haug E, Aaro LE. Measuring mental well-being in Norway: validation of the Warwick-Edinburgh Mental Well-being Scale (WEMWBS). *BMC Psychiatry* 2017; **17**(1): 182.

8. Brown KW, Ryan RM. The benefits of being present: mindfulness and its role in psychological well-being. *J Pers Soc Psychol* 2003; **84**(4): 822-48.

9. Pallesen S, Bjorvatn B, Nordhus IH, Sivertsen B, Hjornevik M, Morin CM. A new scale f or measuring insomnia: the Bergen Insomnia Scale. *Percept Mot Skills* 2008; **107**(3): 691-706.

10. Heart rate variability: standards of measurement, physiological interpretation and clinical use. Task Force of the European Society of Cardiology and the North American Society of Pacing and Electrophysiology. *Circulation* 1996; **93**(5): 1043-65.

11. Berntson G, Bigger J, Eckberg D, et al. Heart rate variability: Origins, methods, and interpretive caveats*. Psychophysiology* (1997); **34**. 623-48.

12. Thayer J F, Jamamoto S S, Brosschot J F. The relationship of autonomic imbalance, heart rate variability and cardiovascular disease risk factors. *Int. J. Cardiol* (2010); **141**(2):122-31

13. Giles D, Draper N, Neil W. Validity of the Polar V800 heart rate monitor to measure RR intervals at rest. *European journal of applied physiology* 2016; **116**(3): 563-71.
